# Trace elements in pancreatic cancer

**DOI:** 10.1002/cam4.7454

**Published:** 2024-07-17

**Authors:** Yao Yanjun, Zhuang Jing, Song Yifei, Gu Gangzhao, Yan Chenxin, Wei Qiang, Yan Qiang, Han Shuwen

**Affiliations:** ^1^ Huzhou Central Hospital, Affiliated Huzhou Hospital Zhejiang University School of Medicine Huzhou China; ^2^ Shulan International Medical school Zhejiang Shuren University Hangzhou China; ^3^ Institut Catholique de Lille, Junia (ICL), Université Catholique de Lille, Laboratoire Interdisciplinaire des Transitions de Lille (LITL) Lille France

**Keywords:** arsenic, copper, manganese, pancreatic cancer, selenium, trace elements

## Abstract

**Background:**

Pancreatic cancer (PCA) is an extremely aggressive malignant cancer with an increasing incidence and a low five‐year survival rate. The main reason for this high mortality is that most patients are diagnosed with PCA at an advanced stage, missing early treatment options and opportunities. As important nutrients of the human body, trace elements play an important role in maintaining normal physiological functions. Moreover, trace elements are closely related to many diseases, including PCA.

**Review:**

This review systematically summarizes the latest research progress on selenium, copper, arsenic, and manganese in PCA, elucidates their application in PCA, and provides a new reference for the prevention, diagnosis and treatment of PCA.

**Conclusion:**

Trace elements such as selenium, copper, arsenic and manganese are playing an important role in the risk, pathogenesis, diagnosis and treatment of PCA. Meanwhile, they have a certain inhibitory effect on PCA, the mechanism mainly includes: promoting ferroptosis, inducing apoptosis, inhibiting metastasis, and inhibiting excessive proliferation.

## 1 INTRODUCTION

Pancreatic cancer (PCA) is an extremely aggressive malignant cancer that is the fourth leading cause of cancer death globally^1^ and the sixth leading cause of cancer death in China.^2^ The incidence of PCA is increasing annually, and its rank among cancer causes of death is constantly increasing.^3^ PCA has a high mortality rate, with a 5‐year survival rate of no more than 10%,^1^ mainly because >80% of patients are already in the advanced stage when they are diagnosed, missing the optimal treatment opportunity.^4^ Improving the diagnosis and treatment status of PCA is the only way to reduce its incidence, increase its early diagnosis rate and improve its therapeutic efficacy.

Risk factors for PCA include smoking, drinking, obesity, diabetes, chronic pancreatitis, and genetics.^5^, ^6^ Trace elements are essential nutrients in the human body and play an important role in normal physiological functions. In addition to dietary intake, the concentration of trace elements in the environment (such as soil, water, and air) will also affect the level of trace elements in the human body. When the concentration of trace elements in the environment is high or low, the body will also increase or decrease absorption. Manganese has strong redox activity and plays an important role in biological processes such as energy metabolism, antioxidant activity, detoxification, musculoskeletal growth, and brain and nerve function regulation.^7^ Zinc is involved in cell differentiation, division, growth, transport, and gene expression^8^ and is involved in more than 2000 transcription factors and 300 enzymes.^9^ Copper is involved in signaling pathways such as active oxygen metabolism, cellular energy metabolism, iron absorption, and signal transduction.^10^ While maintaining normal physiological functions, trace elements are closely related to certain diseases in the human body. Low levels of selenium in the body may increase the risk of cardiovascular disease, inflammatory bowel disease^11^ and pregnancy complications.^12^ Long‐term exposure to manganese can increase the risk of Parkinson's disease,^13^ which has been validated in animal experiments.^14^ The serum selenium concentration in diabetic patients is higher than that in healthy individuals.^15^ However, serum concentrations of zinc and iron in obese women are lower than those in healthy individuals.^16^

In addition, trace elements also play important roles in tumors. Serum strontium concentration in breast cancer patients is higher than that in healthy individuals.^17^ Gastric cancer tissues contain higher concentrations of iron, magnesium, and arsenic than noncancerous tissues.^18^ Colorectal cancer tissues have higher concentrations of zinc, chromium, copper, aluminum and lead than noncancerous tissues.^19^ A controlled study involving 118 patients with PCA and 399 healthy individuals showed that high concentrations of cadmium, arsenic and lead significantly increase the risk of PCA, while high concentrations of selenium and nickel are negatively correlated with the risk of PCA.^20^

In addition to the risk of tumors, the relevant mechanisms of trace elements in the development and treatment of tumors have also been studied. Arsenic may play a carcinogenic role through the PI3K/AKT/mTOR pathway, EGFR,^21^ Ras/Raf/MEK/ERK^22^ and Nrf2.^23^ Copper plays a key role in cancer initiation signaling pathways, such as the MAPK–ERK,^24^ P38 MAPK,^25^ ULK^26^ and PDK1‐AKT pathways.^26^ MnCl2, an agonist of cGAS‐STING, promotes the production of type I interferon,^27^ manganese molybdate nanoparticles reduce the expression of glutathione in tumors,^28^ and MnO_2_ promotes the production of reactive oxygen species (ROS),^29^ all of which can promote the ferroptosis of tumor cells. Zinc can inhibit tumor growth by negatively regulating NF‐κB transcription factor activity and exerting antioxidant effects.^30^ The antitumor effects of selenium may involve the following mechanisms: downregulating hypoxia‐inducing factors, inhibiting tumor angiogenesis,^31^ promoting DNA repair,^32^ and inhibiting tumor cell proliferation.^33^

**FIGURE 1:**
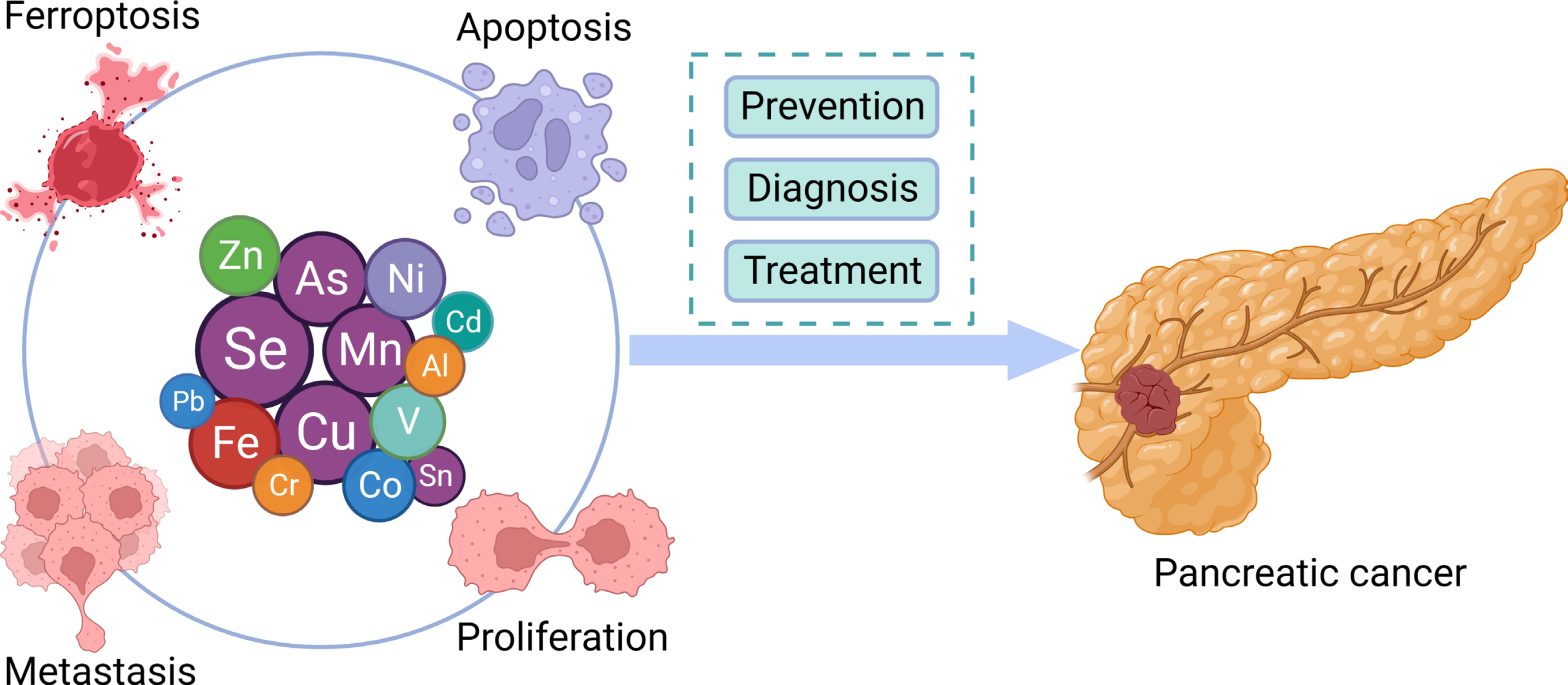
Association between trace element levels and pancreatic cancer incidence.

Trace elements are strongly linked to metabolic diseases such as diabetes and obesity, and they are risk factors for PCA. Differences in trace element levels between PCA patients and healthy individuals have also been reported. These findings all prove that there is a close association between trace elements and PCA (Figure 1). This review summarized the associations between different trace elements and the risk of PCA (Table 1), summarized the anti‐pancreatic cancer mechanism involving trace elements, aimed to clarify the application of trace elements in PCA, and provided a new reference for the prevention, diagnosis, and treatment of PCA.

**TABLE 1:** The concentrations of trace elements with PCA.

Element	Index	Sample size	Results	Reference
Selenium	Intake	Case = 1424	Selenium intake was negatively associated with the risk of PCA (RR = 0.659, 95% CI = 0.489–0.889).	37
Selenium	Intake	/	Selenium intake was negatively associated with the risk of PCA (OR = 0.47, 95% Cl = 0.26–0.85).	38
Selenium	Intake	Case = 184	Selenium intake was negatively associated with the risk of PCA (HR = 0.58, 95% CI = 0.35–0.94).	39
Selenium	Intake	Case = 49	Combined intake of vitamins C, E, and selenium was associated with a lower risk of PCA (HR = 0.33, 95% CI = 0.13–0.84).	40
Selenium	Serum	Case = 100 Control = 100	The serum selenium concentration of PCA was significantly lower than healthy individuals (*p* < 0.0001).	41
Selenium	Toenail	Case = 118 Control = 399	High concentration of selenium in toenails was negatively associated with the risk of PCA (OR = 0.05, 95% CI = 0.02–0.15).	20
Selenium	Pancreatic juice	Case = 35 Control = 35	The concentration of selenium in pancreatic juice of PCA was higher than that of healthy individuals (*p* = 0.01).	42
Selenium	Serum	Case = 303 Control = 606	Serum selenium concentration was not significantly associated with the risk of PCA (OR = 0.66, 95% CI = 0.32–1.37).	43
Copper	Serum	Case = 100 Control = 100	The serum copper concentration of PCA was significantly higher than that of healthy individuals (*p* < 0.0001).	41
Copper	Urine	Case = 21 Control = 46	The concentration of copper in urine of PCA was significantly higher than that of healthy individuals (*p* < 0.05).	57
Arsenic	Toenail	Case = 118 Control = 118	High arsenic concentration in toenails was positively associated with the risk of PCA (OR = 2.02, 95% CI = 1.08–3.78).	20
Arsenic	Toenail	Case = 78 Control = 416	There was no significant difference in arsenic concentration in toenail between PCA and healthy individuals (*p* > 0.05).	84
Arsenic	Intake	Case = 11,405	Arsenic intake increased risk of PCA (OR = 2.1, 95% CI = 1.9–2.4).	85
Manganese	Toenail	Case = 23 Control = 91	PCA with occupational exposure to Ahs had higher concentration of manganese in their toenails (*p* < 0.05).	111
Manganese	Toenail	Case = 78 Control = 416	The concentration of manganese in toenails of K‐ras mutant PCA was significantly lower than that of healthy individuals (*p* < 0.05).	84
Iron	Toenail	Case = 78 Control = 416	The concentration of iron in toenails of Kras wild‐type PCA was higher than that of Kras mutant (*p* < 0.05).	84
Iron	Toenail	Case = 23 Control = 91	PCA with occupational exposure to Ahs had higher concentration of iron in their toenails, but there was no statistical significance (*p* > 0.05).	111
Zinc	Intake	Case = 1659	Zinc intake was negatively associated with the risk of PCA (RR = 0.798, 95% Cl = 0.621–0.984).	134
Cadmium	Toenail	Case = 118 Control = 399	Cadmium concentration in toenails was positively associated with the risk of PCA (OR = 3.58, 95% CI =1.86–6.88).	20
Cadmium	Toenail	Case = 78 Control = 416	The concentration of cadmium in toenails of PCA was significantly higher than that of healthy individuals (*p* < 0.01).	84
Cadmium	Tissue	Case = 31 Control = 29	Increased concentration of cadmium in the pancreas was associated with increased risk of PCA (OR = 3.200, 95% Cl = 1.051–9.473).	138
Lead	Toenail	Case = 118 Control = 399	Lead concentration in toenails was positively associated with the risk of PCA (OR = 6.26, 95% CI = 2.71–14.47).	20
Nickel	Toenail	Case = 118 Control = 399	Nickel concentration in toenails was negatively associated with the risk of PCA (OR = 0.27, 95% CI = 0.12–0.59).	20
Nickel	Toenail	Case = 78 Control = 416	The concentration of arsenic in toenails of K‐ras mutant PCA was significantly lower than that of healthy individuals (*p* < 0.01).	84
Chromium	Pancreatic juice	Case = 35 Control = 35	Chromium concentration in pancreatic juice were positively associated with the risk of PCA (OR = 3.1,95 % Cl = 1.23–7.8).	42
Vanadium	Toenail	Case = 23 Control = 91	PCA with occupational exposure to Ahs had higher concentration of vanadium in their toenails (*p* < 0.05).	111

Abbreviations: Ahs, aromatic solvents; PCA, pancreatic cancer.

## 2 SELENIUM

Selenium usually exists in nature in the oxidation states of −2, 0, +4, and + 6. In living organisms, selenium exists in two forms, one as selenium and the other as a component of various compounds (such as selenates, selenites, selenomethionine, selenocysteine, and proteins containing these amino acids). Selenium affects the activation and differentiation of macrophages, NK cells, B cells, and T cells, mainly by stimulating the antibody formation and activity of helper T cells, cytotoxic T cells and NK cells.^34^ At the same time, the increase of selenium intake will increase the abundance of *lactobacillus* in the gut.^35^ The human body contains approximately 14–21 mg of selenium, which is mainly distributed in the liver, pancreas, kidney, spleen, tooth enamel, and nails. Selenium‐rich plants are the main source of selenium in the human diet, including selenium‐rich rice, selenium‐rich wheat, mushrooms, garlic, black sesame, black beans, etc. The recommended intake of selenium for adults is 70‐200 μg day^−1^, and organic selenium is healthier and safer than inorganic selenium.^36^ After ingestion, selenium is mainly absorbed in the duodenum and small intestine, reaches the liver through the portal vein, and is subsequently transported to peripheral tissues through the blood circulation with the selenium protein as a carrier. The liver regulates the amount of selenium in the body, and excess selenium is excreted mainly through the urine and feces. Excessive accumulation of selenium in the body is rare and is mainly seen in drinking water with high selenium content, or in certain jobs where there is frequent exposure to selenium. The main manifestations of excessive selenium accumulation are hair loss, nail abnormalities, and dermatitis.

**FIGURE 2:**
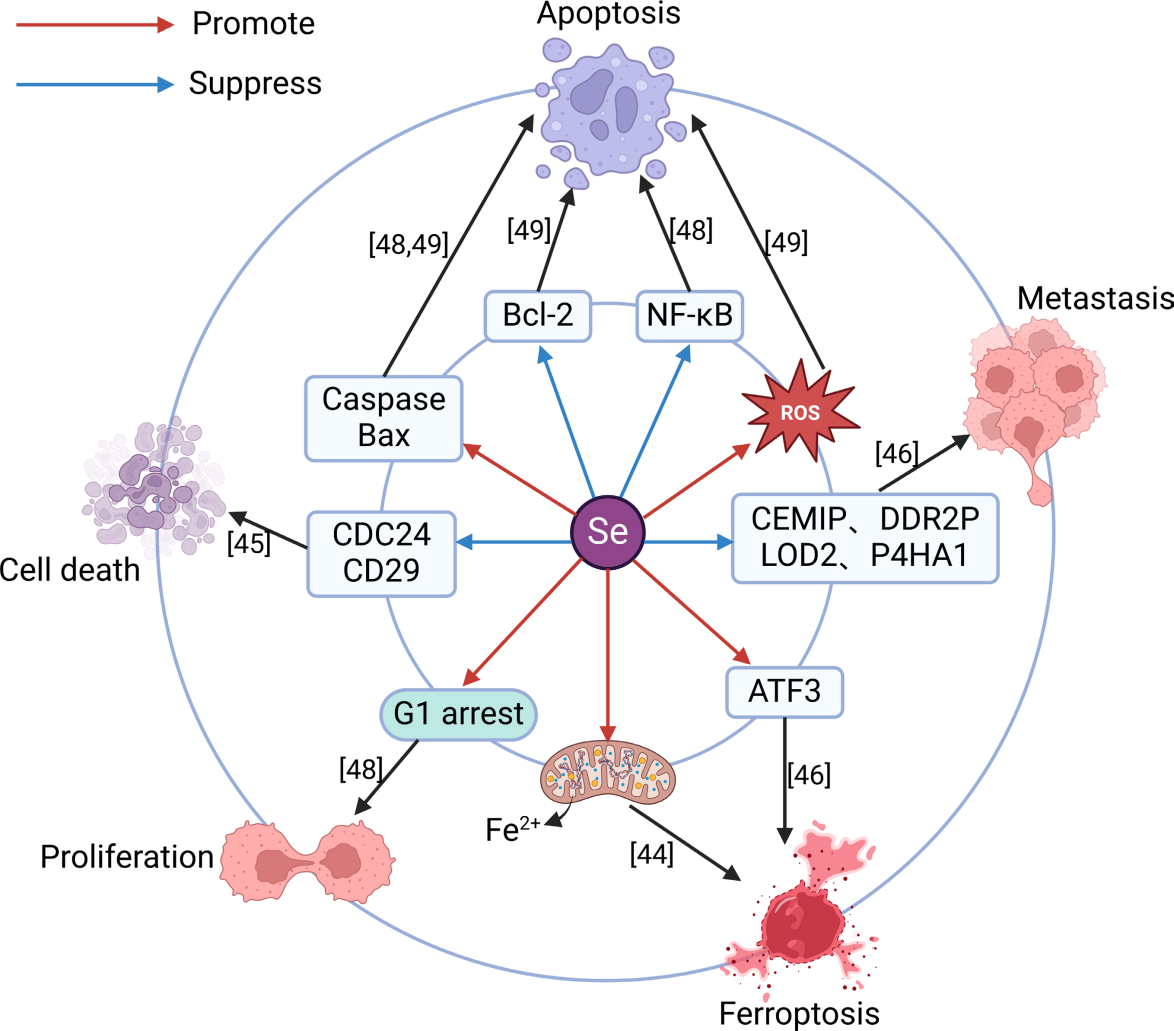
Mechanism of selenium in anti‐pancreatic cancer. Selenium is involved in APCA through inducing apoptosis, promoting ferroptosis, inhibiting metastasis, and inhibiting excessive proliferation. ATF3, activating transcription Factor 3; Bax, Bcl2‐associated x; Caspase, cysteinyl aspartate specific proteinase; CD29, β1‐integrin; CDC24, cell division control protein 42 homolog; CEMIP, cell migration inducing hyaluronic binding protein; DDR2, discoidin domain receptor 2; NF‐κB, nuclear factor κB; P4HA1, prolyl 4‐hydroxylase alpha polypeptide 1; PLOD2, procollagen‐lysine,2‐oxoglutarate 5‐dioxygenase 2; ROS, reactive oxygen species.

The association between selenium and PCA has been extensively studied. A meta‐analysis of 1424 patients with PCA^37^ and a meta‐analysis of 6 studies suggested that higher selenium intake may decrease the risk of PCA^38^; these results may be related to the use of selenium as a dietary antioxidant.^39^, ^40^ A retrospective study indicated that reduced serum selenium concentrations were associated with an increased risk of PCA, and high concentrations of selenium in patients with PCA were associated with prolonged survival.^41^ A controlled study involving 118 patients with PCA and 399 healthy individuals revealed that high concentrations of selenium were negatively correlated with the risk of PCA.^20^ Taken together, these findings indicate that high concentrations of selenium can decrease the risk of PCA. However, some scholars came to the opposite conclusion. After the concentrations of selenium in the pancreatic juice of patients with PCA, patients with chronic pancreatitis and healthy individuals were measured, it was found that the concentration of selenium in pancreatic juice was strongly correlated with the risk of PCA. Compared with that of healthy individuals, the risk of PCA was 160% greater for every 1 SD increase in the selenium concentration and 480% greater for every 1 SD increase in the sum of the chromium and selenium concentrations.^42^ Interestingly, the serum selenium concentrations of 303 patients with PCA and 606 healthy individuals were analyzed, and no significant correlation was found between the serum selenium concentration and the risk of PCA.^43^ These findings led to a contradiction between the previous association between the selenium concentration in the body and the risk of PCA, and additional studies are needed to provide additional evidence. Current studies have indicated that high concentrations of selenium can decrease the risk of PCA, and the underlying mechanism has been extensively explored (Figure 2). Organic selenium can release iron from mitochondria to increase the sensitivity of pancreatic cancer cells (PCACs) to ferroptosis, thus inhibiting tumor growth.^44^ A new mechanism for the toxicity of novel methylselenoesters on PCACs was proposed: downregulating cell division control protein 42 homolog (CDC42) and its downstream effector β1‐integrin (CD29) to induce cell segregation, leading to inner cell formation and inner cell death.^45^ Sodium selenite decreases the expression of genes that promote PCA metastasis (CEMIP, DDR2, PLOD2, and P4HA1) and increases the expression of the ATF3 gene, which promotes ferroptosis in PCACs, thereby specifically reducing the activity of PCACs and inhibiting tumor growth.^46^ NF‐κB is involved in the development of PCA and inhibits the apoptosis of PCACs.^47^ A novel selenoaspirinyl compound that can induce caspase‐mediated apoptosis and G1 cell cycle arrest inhibits NF‐κB signaling to inhibit PCACs proliferation, and it can enhance the cytotoxicity of gemcitabine to PCA.^48^ As photosensitizing agents for photodynamic therapy, CdSe/ZnS quantum dots increase ROS production in PCACs, upregulate Bax, and caspase‐3 and downregulate Bcl‐2 to induce apoptosis in PCACs, thus inhibiting the proliferation of PCA.^49^ In summary, the anti‐pancreatic cancer mechanisms associated with selenium mainly include promoting apoptosis, promoting ferroptosis, inhibiting metastasis, and inhibiting excessive proliferation.

In addition, selenium has also shown great application value in the treatment of PCA. A study showed that the combination of selenium and gemcitabine has a synergistic anticancer effect on BxPC‐3 cells,^50^ and selenium enhances the anticancer activity of gemcitabine. Selenium participated in the formation of a carrier named Abraxane@MoSe, which can accurately guide local photothermal chemotherapy in vivo, reduce cancer‐related fibroblasts, and enhance the synergistic treatment efficacy of PCA.^51^ A biocompatible near‐infrared fluorescence probe called glucose‐functionalized Ag2Se QDs, which can target the imaging of PCA,^52^ is highly valuable for the diagnosis and accurate treatment of PCA. A study of 271 patients (including PCA) revealed that selenium deficiency was common in PCA patients and that selenium supplementation improved overall quality of life, physical and emotional functions, and fatigue.^53^ Therefore, selenium supplementation may improve the prognosis of PCA patients.

## 3 COPPER

Copper is present in the human body mainly as ceruloplasmin, which plays an important role in electron transport and redox reactions. It can cause the pro‐activation state of Th1 and Th2 lymphocytes^54^ and promote the pro‐inflammatory state of macrophages.^55^ In addition, high levels of copper intake promote the development of antimicrobial resistance in the gut microbiota.^56^ The adult body contains approximately 0.1 g of copper, 50%–70% of which is present in muscle and bone, 20% in the liver, and 5%–10% in the blood; moreover, the amount of copper distributed in the pancreas is lower. Dietary copper is the main source of copper in the human body, including beef liver, oysters, mushrooms, cashews, sunflower seeds, potatoes, etc. After oral intake, copper‐containing food is mainly absorbed in the duodenum, reaches the liver for metabolism through the portal vein system, and then is distributed throughout the whole body through the blood circulation. Copper‐transporting ATPases maintains intracellular copper levels, and its inactivation leads to intracellular copper accumulation and excretion disorders. Copper is excreted into the body mainly through bile in the intestine and is subsequently excreted in the form of feces.

**FIGURE 3:**
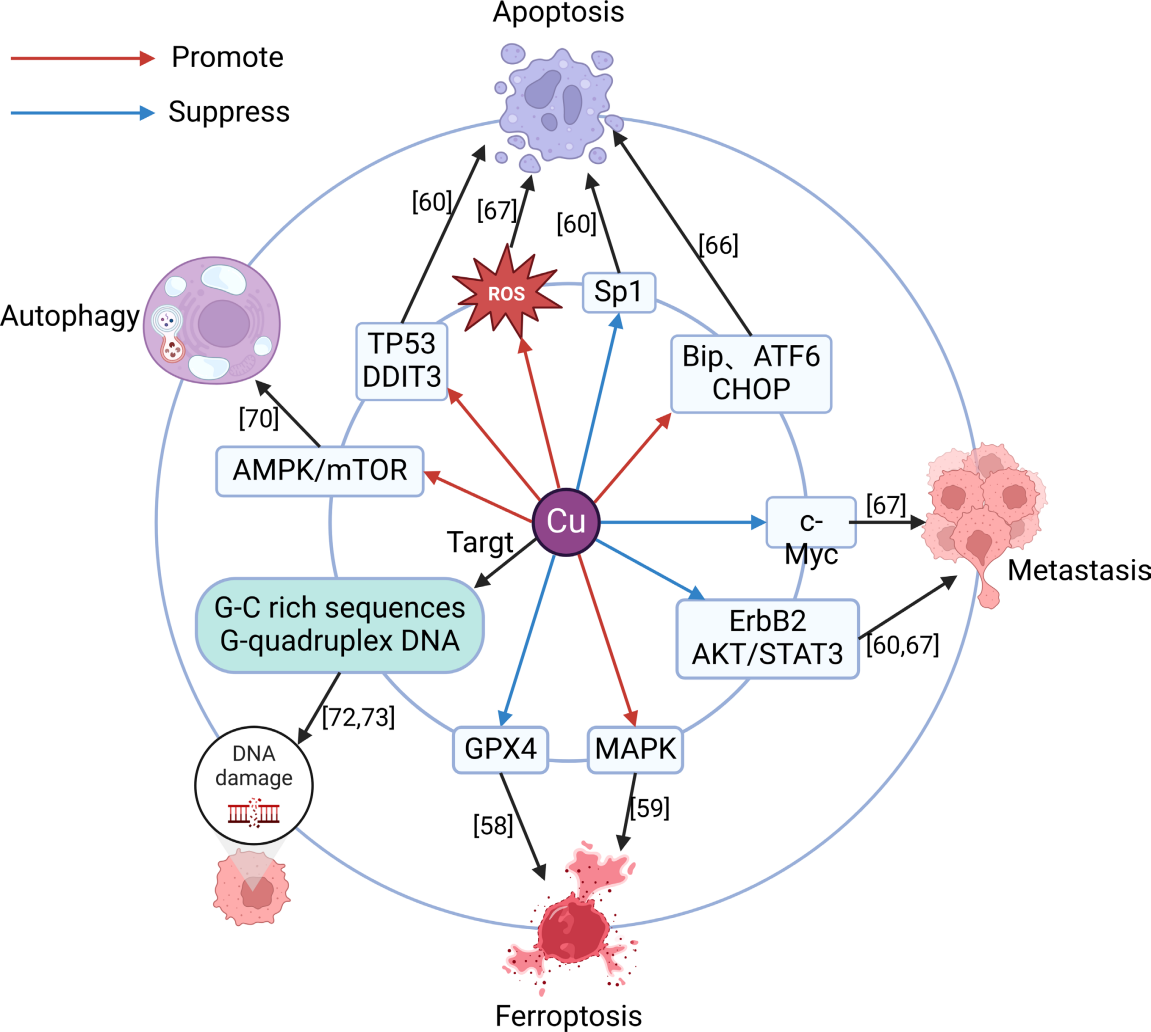
Mechanism of copper in anti‐pancreatic cancer. Copper is involved in APCA through promoting apoptosis, promoting ferroptosis, inhibiting metastasis, promoting autophagy, and targeting tumor DNA damage.AKT/STAT3, serine/threonine kinase/signal transducer and activator of transcription 3 signaling pathway; AMPK/mTOR, adenosine monophosphate‐activated protein kinase/mammalian target of rapamycin signaling pathway. ATF6, activating transcription Factor 6; Bip, heavy chain‐binding protein; CHOP, c/ebp homologous protein; DDIT3, DNA damage inducible transcript 3; GPX4, glutathione peroxidase 4; MAPK, mitogen‐activated protein kinase signaling pathway; ROS, reactive oxygen species; Sp1, specificity protein 1.

High concentrations of copper may increase the risk of PCA. A comparison of the serum copper concentrations between 100 patients with PCA and 100 healthy individuals showed that patients with PCA had higher serum copper concentrations.^41^ A study involving urine samples from 67 individuals showed that patients with PCA had higher concentrations of copper in their urine.^57^ Similarly, the mechanism of action of copper in treating pancreatic cancer has also been extensively studied **(**Figure 3 **)**. Exogenous copper can bind to glutathione peroxidase 4 (GPX4) cysteines C107 and C148 to increase GPX4 ubiquitination and the formation of GPX4 aggregates, subsequently enabling GPX4 autophagy to enhance ferroptosis in PCACs,^58^ thus playing a role in PCACs. [CuII2I23]3 also exhibited anti‐pancreatic cancer (APCA) activity by activating the MAPK pathway to promote ferroptosis.^59^ A study noted that copper‐tolfenamic acid could inhibit the growth of PCACs and that the expression of TP53 and DDIT3 increased, while the expression of Sp1, ErbB2, and STAT3 decreased.^60^ TP53 is a common tumor suppressor gene that can promote the apoptosis of cancer cells.^61^ Like TP53, DDIT3 can also promote the apoptosis of cancer cells.^62^ Sp1 inhibits apoptosis.^63^ ErbB2 is a common oncogene^64^ that can activate AKT/STAT3 to promote the occurrence and metastasis of PCA.^65^ The heteroleptic Cu(II)‐phenanthroline‐UDCA upregulates the chaperone BiP, the proapoptotic protein CHOP, and the transcription factor ATF6 to promote apoptosis.^66^ The Cu(II)‐cyamonin complex can produce ROS to induce apoptosis, inhibit the AKT signaling pathway and downregulate c‐Myc to inhibit the growth of PCACs and metastasis.^67^ As mentioned above, the AKT signaling pathway promotes the metastasis of PCA, while the main carcinogenic mechanism of c‐Myc is to promote cell proliferation.^68^ Cu(I) and Cu(II) complexes based on lonidamine‐conjugated ligands can also induce PCACs death through apoptosis.^69^ Zinc‐doped copper oxide nanocomposites induced autophagy in PCACs by activating the AMPK/mTOR pathway,^70^ which led to the death of PCACs. Roy J et al. noted that cu can enhance the cytotoxicity of quinolyl pyrazinamides to PCACs,^71^ but the specific mechanism involved remains to be studied. Polypyridyl‐based copper phenanthrene complexes can specifically recognize guanine‐cytosine (G‐C)‐enriched sequences and cause cytotoxicity in PCA.^72^ Copper(ii)l/d‐valine‐(1,10‐phen) complexes effectively target telomere G‐quadruplexes and undergo oxidative cleavage, demonstrating cytotoxicity to PCA.^73^ Copper promotes APCA activity mainly through promoting apoptosis, promoting ferroptosis, inhibiting metastasis, promoting autophagy, and targeting tumor DNA damage.

Copper also has great potential in the diagnosis and orientation of PCA. Injection of copper‐64‐labeled drugs can improve the sensitivity of PET‐CT for diagnosing PCA and can detect tumors with a minimum diameter of 3 mm.^74^ This achievement can be said of a large step forward in the early detection of PCA. Copper‐doped nanomaterials can also be used as drug carriers to enhance the killing effect of drugs on PCA through specific targeted drug delivery^73^, ^75^, ^76^, ^77^ and improved drug tumor penetration.^78^ Interestingly, copper chelating agents could silence CTR1 and downregulate the phosphorylation of AKT/mTOR molecules after reducing copper concentrations, thus inhibiting PCA progression and improving the anticancer effect of rapamycin on PCA.^79^, ^80^ These findings further confirmed that copper homeostasis can be used as an emerging target for treatment of PCA. Copper plays an important role in the discovery, diagnosis and treatment of PCA, but different concentrations of copper have different effects on PCA, and maintaining copper homeostasis is crucial for ensuring the health of the pancreas.

## 4 ARSENIC

Arsenic has been identified as a carcinogen by the International Agency for Research on Cancer and has a definite carcinogenic effect on the human body. Arsenic is also an immunotoxicant that promotes the anti‐inflammatory phenotype of macrophages, inhibits T cell proliferation, B cell proliferation, and antibody production.^81^ Moreover, long‐term exposure to arsenic can also reduce the abundance of *Clostridiaceae, Rikenellaceae*, and *Parabacteroides* in the gut.^82^ Arsenic exists in nature mainly in the form of inorganic arsenic and is widely distributed in soil, water, and air. Arsenic is absorbed by the body through exposure to an environment contaminated with arsenic, mainly through inhalation or oral absorption. Drinking water containing arsenic is the most common form of arsenic. When absorbed by the body, arsenic binds to erythrocytes and is deposited in the liver, kidneys, muscles, bones, hair, skin, and nails through the blood circulation, where it can cause toxicity. While arsenic is harmful to the kidneys, it is also excreted in the urine.

Several studies have pointed to a strong association between the concentrations of arsenic in the body and the risk of PCA. The concentrations of trace elements in the toenails of 118 patients with PCA and 399 healthy individuals were analyzed, and it was found that high concentrations of arsenic can increase the risk of PCA.^20^ A study involving 78 patients with PCA and 416 healthy individuals indicated that patients with PCA had higher concentrations of arsenic in their toenails, but the difference was not statistically significant.^83^ A cohort study of 11,405 patients with PCA indicated that long‐term drinking of water contaminated with arsenic increased the risk of PCA.^84^

**FIGURE 4:**
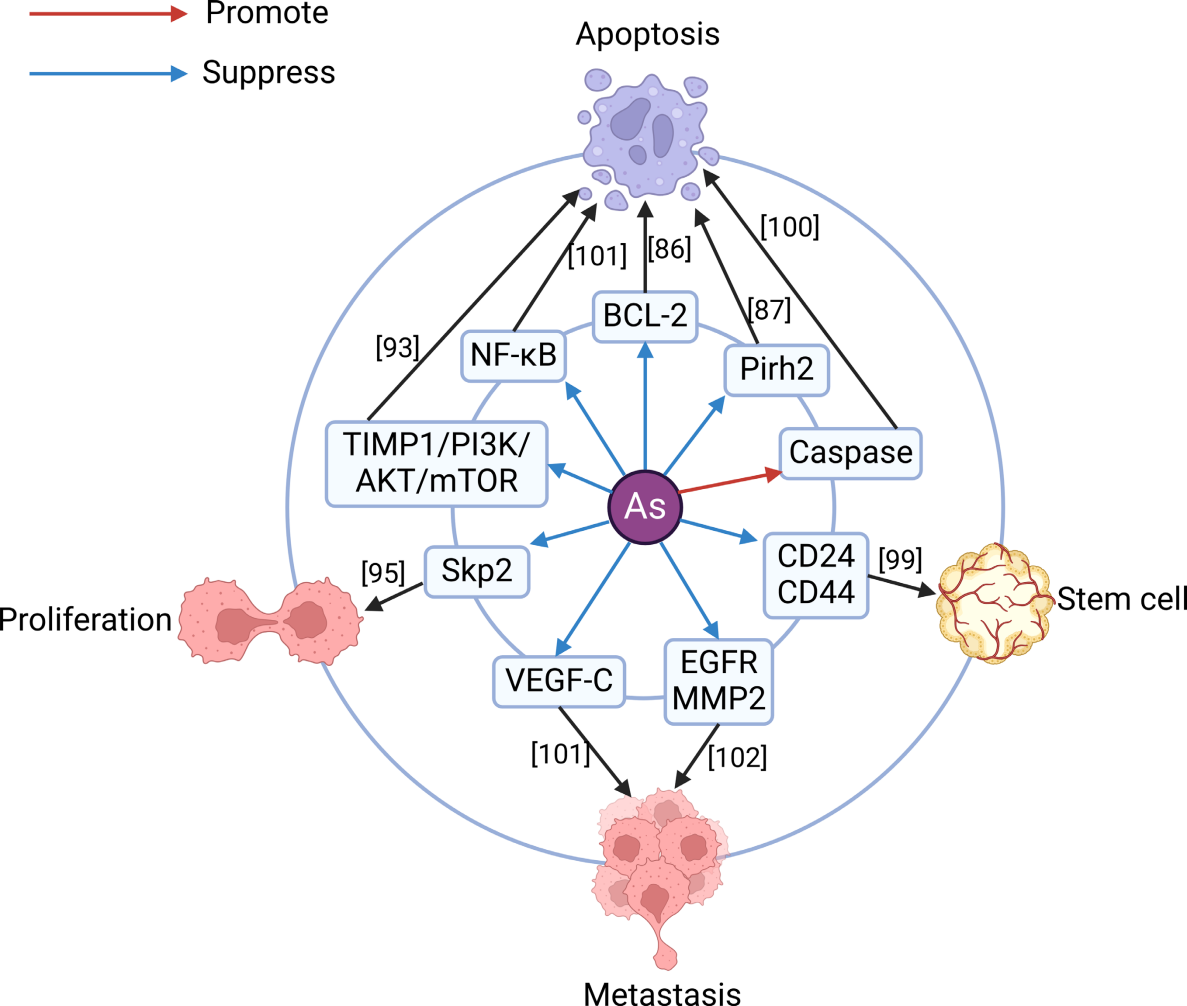
Mechanism of arsenic in anti‐pancreatic cancer. Arsenic is involved in APCA through promoting apoptosis, inhibiting metastasis, and inhibiting excessive proliferation. Caspase, cysteinyl aspartate specific proteinase; CD24, innate immunosuppressive factor; CD44, cell surface adhesion molecule; EGFR, epidermal growth factor receptor; MMP2, matrix metalloproteinase 2; NF‐κB, nuclear factor κB. Pirh2, p53‐dependent ubiquitin ligase; Skp2, S‐phase kinase‐associated protein 2; TIMP1/PI3K/AKT/mTOR, tissue inhibitor of matrix metalloproteinases 1/phosphoinositide 3‐kinase/serine/threonine kinase/mammalian target of rapamycin signaling pathway; VEGF‐C, vascular endothelial growth factor‐C.

Interestingly, the poison arsenic has shown strong potential in the treatment of PCA, and its mechanism involves mainly promoting apoptosis, inhibiting metastasis, and inhibiting excessive proliferation (Figure 4). Arsenic promoted the apoptosis of PCACs cells by downregulating BCL‐2.^85^ A study showed that both arsenic trioxide (ATO) and pyrrolidine dithiocarbamate‐arsenic trioxide (PDTC‐ATO) can promote apoptosis to inhibit PCACs, and the inhibitory effect of PDTC‐ATO is stronger than that of ATO^86^; moreover, both can downregulate Pirh2. Pirh2 can downregulate the expression of P53,^87^ thereby inhibiting apoptosis and causing adverse effects.^61^ The BET bromodomain inhibitor JQ1,^88^ heme oxygenase‐1,^89^ hypoxia‐inducing factor‐1 inhibitor^90^ and docosahexaenoic acid^91^ can all synergistically enhance ATO‐induced apoptosis in PCACs. In addition to inhibiting the proliferation of PCACs, ATO can also enhance the sensitivity of PCACs to gemcitabine by downregulating TIMP1/PI3K/AKT/mTOR signaling,^92^ inhibiting the activation of pancreatic stellate cells,^93^ and inhibiting the expression of Skp2.^94^ TIMP1/PI3K/AKT/mTOR has an inhibitory effect on apoptosis,^92^ while Kp2 has a carcinogenic effect by activating Myc and causing cell proliferation.^95^ ATO, while increasing the sensitivity to chemical drugs, can also play an APCA role through multiple mechanisms. In addition to gemcitabine, ATO can also overcome the resistance of PCACs to erlotinib,^96^ thereby enhancing the effect of erlotinib on APCA. ATO also functions as a sensitizer in radiotherapy for PCA,^97^ and its mechanism may involve reversing the hypoxic environment and downregulating CD24 and CD44. Coincidentally, CD24 and CD44 have been identified as being characteristically expressed in PCA stem cells.^98^ ATO plays an important role both in direct APCA and in improving therapeutic efficacy, and it has great prospects in the treatment of PCA. In addition to ATO, arsenic sulfide also promoted APCA activity, which may be achieved by activating caspase‐3 and caspase‐9 to induce apoptosis.^99^ Sodium meta‐arsenite inhibited metastasis by inhibiting NF‐κB to promote apoptosis and inhibit VEGF‐C activity,^100^ and it can enhance the anticancer activity of gemcitabine against PCA by downregulating EGFR and MMP2.^101^ EGFR^102^ and MMP2^103^ both play important roles in promoting the growth and metastasis of PCACs. Oxidative phosphorylation inhibitors can inhibit the recurrence of KRAS‐mutant PCA,^104^ and ATO, an oxidative phosphorylation inhibitor,^105^ may inhibit the recurrence of PCA. Arsenic has shown strong APCA effects, but considering its own toxic effects, its safety in practical applications remains to be studied.

## 5 MANGANESE

Manganese is usually found in the natural positive oxidation state (+2, +3, +4, +6, and + 7); the most common oxidation states in living organisms are +2 and + 3, which are involved in the composition of a variety of coenzymes in the body and play key roles in protecting cells from damage by the superoxide free radical O2−. Moreover, manganese can also act as an immune enhancer, stimulating humoral and cellular immune responses, inducing antibody production and CD4/CD8T cell proliferation and activation.^106^ Manganese exposure also has an effect on microbiota, it can significantly reduce *Bacteroides* in the gut of male mice.^107^ Interestingly, there is a high content of *Bacteroidetes* in human PCA tissues, which may promote the development of PCA by inducing immunosuppression.^108^ But, whether manganese inhibits PCA by reducing the abundance of *Bacteroidetes* needs further study. In the human body, manganese comes from food intake (such as nuts, whole grains, and legumes), inhalation, and skin penetration; is rapidly absorbed in the gastrointestinal tract and lungs; and is subsequently distributed through the blood circulation to soft tissues (~60%), liver (30%), pancreas (5%), kidney (5%), bone (0.5%), and brain (0.1%). Excess manganese is mainly combined with bile in the liver and subsequently excreted in the feces.^109^

**FIGURE 5:**
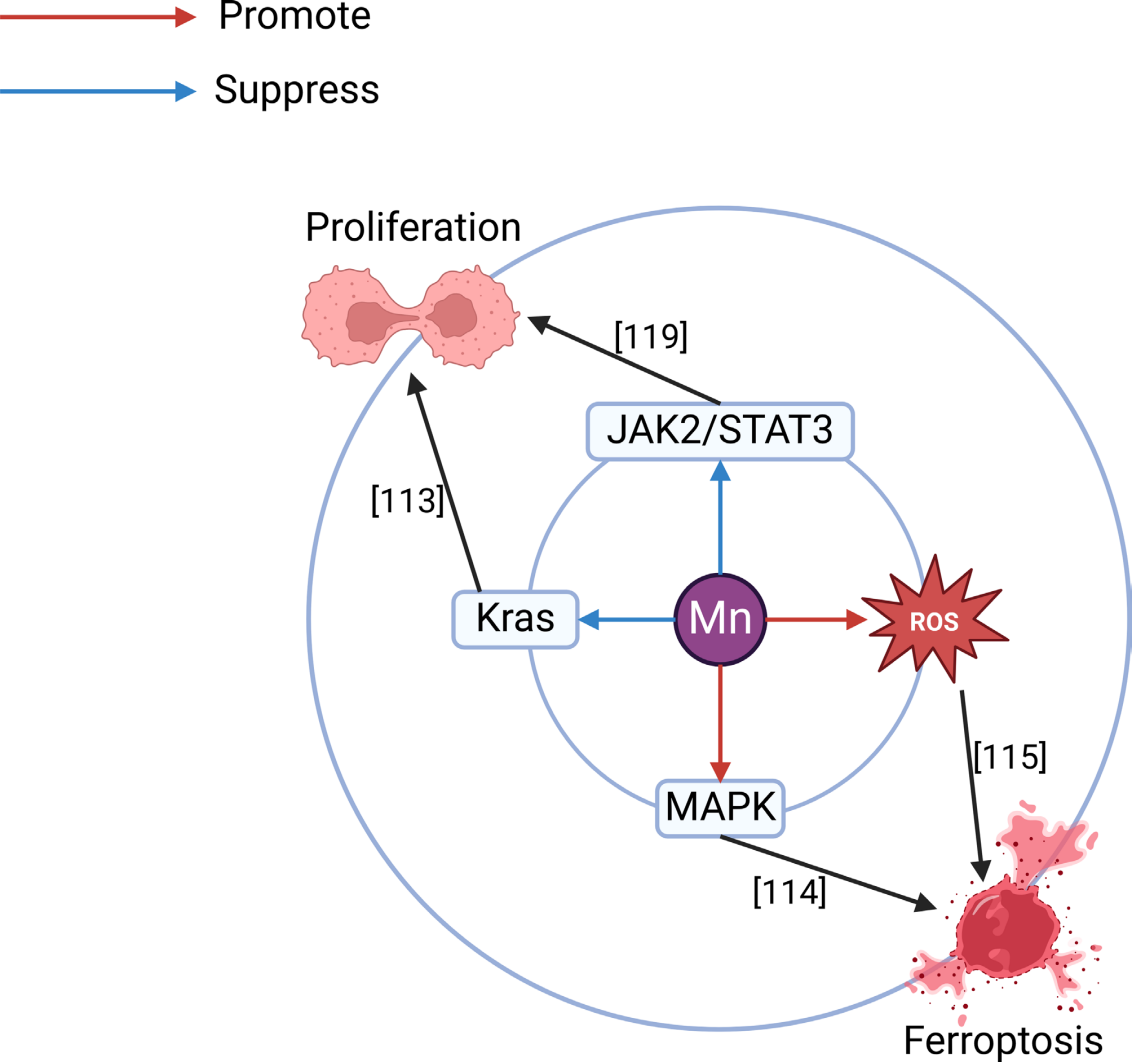
Mechanism of manganese in anti‐pancreatic cancer. Manganese is involved in APCA through mainly promoting ferroptosis and inhibiting excessive proliferation. JAK2/STAT3, Janus kinase 2/signal transducer and activator of transcription 3 signaling pathway; MAPK, mitogen‐activated protein kinase signaling pathway; ROS, reactive oxygen species.

Several studies have shown the close association between manganese and PCA. A study involving 114 patients with PCA showed that the concentration of manganese in the toenails of patients with PCA who were occupationally exposed to aromatic solvents (Ahs) was significantly greater than that of patients who were not exposed to Ahs,^110^ suggesting that manganese combined with Ahs may increase the risk of PCA. A controlled study involving 78 patients with PCA and 416 healthy individuals showed the opposite conclusion: a lower concentration of manganese in toenails was associated with a greater risk of KRAS‐mutant PCA.^83^ Individuals are prone to diabetes when they have both low and high manganese concentrations, and there is a U‐shaped correlation between diabetes and manganese concentrations in the body.^111^ Diabetes is a high‐risk factor for PCA.^5^ These findings suggest that manganese homeostasis is essential for maintaining a healthy state in the pancreas. At present, additional research on the mechanism underlying the interaction between manganese and PCA has focused on APCA (Figure 5). In cell experiments, researchers used manganese‐doped zinc selenide quantum dots for gene delivery and found that they could induce sequence‐specific silencing of carcinogenic Kras mutations in PCA,^112^ thus inhibiting the occurrence of PCA. Manganese‐based Prussian blue nanoparticles induced ferroptosis of PCA through the MAPK pathway,^113^ while PH‐sensitive PtMn nanoparticles could specifically recognize PCACs and increase the production of intracellular ROS to enhance ferroptosis,^114^ thus inhibiting the proliferation and metastasis of PCA. These results indicate that nanoparticles composed of manganese can promote ferroptosis in various ways and exhibit the APCA effect. Manganese superoxide dismutase (MnSOD) had a low expression in PCA,^115^ in other words, high expression of MnSOD can inhibit the occurrence and development of PCA, and it is coincidental that both metformin^116^ and pterostilbene^117^ can increase the expression and activity of MnSOD to inhibit PCA. Liu F et al. introduced galangin into a Fenton‐like catalyst (SiO2@MnO2) to form hybrid nanomedical drugs in response to TME stimulation and confirmed in vivo and in vitro that the nanomedical drug can stimulate intracellular ROS concentrations and inhibit the JAK2/STAT3 cell proliferation pathway to exert APCA effects.^118^ The present study showed that manganese plays an APCA role mainly by inducing ferroptosis and inhibiting the cell proliferation pathway, and additional studies are needed to uncover the APCA mechanism of manganese.

Manganese also plays an important role in the treatment of PCA. Although manganoporphyrins can enhance the toxic effect of gemcitabine on PCA,^119^ they can also enhance the inhibitory effect of ascorbate on PCA.^120^ MnO_2_ can promote the multisource production of ROS and enhance the effectiveness of photodynamic therapy and photothermal therapy.^121^ The manganese complex plays a role as a radiosensitizer^122^ and can also enhance the effect of radiotherapy by dual targeting mechanisms, namely, ameliorating hypoxia and inhibiting angiogenesis.^123^ Avasopasem manganese can increase the concentration of hydrogen peroxide in PCACs, reduce damage to normal cells during radiotherapy, and promote stereotactic radiotherapy of tumors.^124^ Moreover, manganese porphyrin can produce powerful ROS under ultrasonic exposure to enhance the efficacy of sonodynamic therapy for PCA.^125^ At present, manganese has shown potential therapeutic effects in chemical, radiotherapy, photodynamic therapy, photothermal therapy and acoustodynamic therapy, and manganese is of great value in the treatment of PCA.

## 6 OTHER TRACE ELEMENTS

**FIGURE 6:**
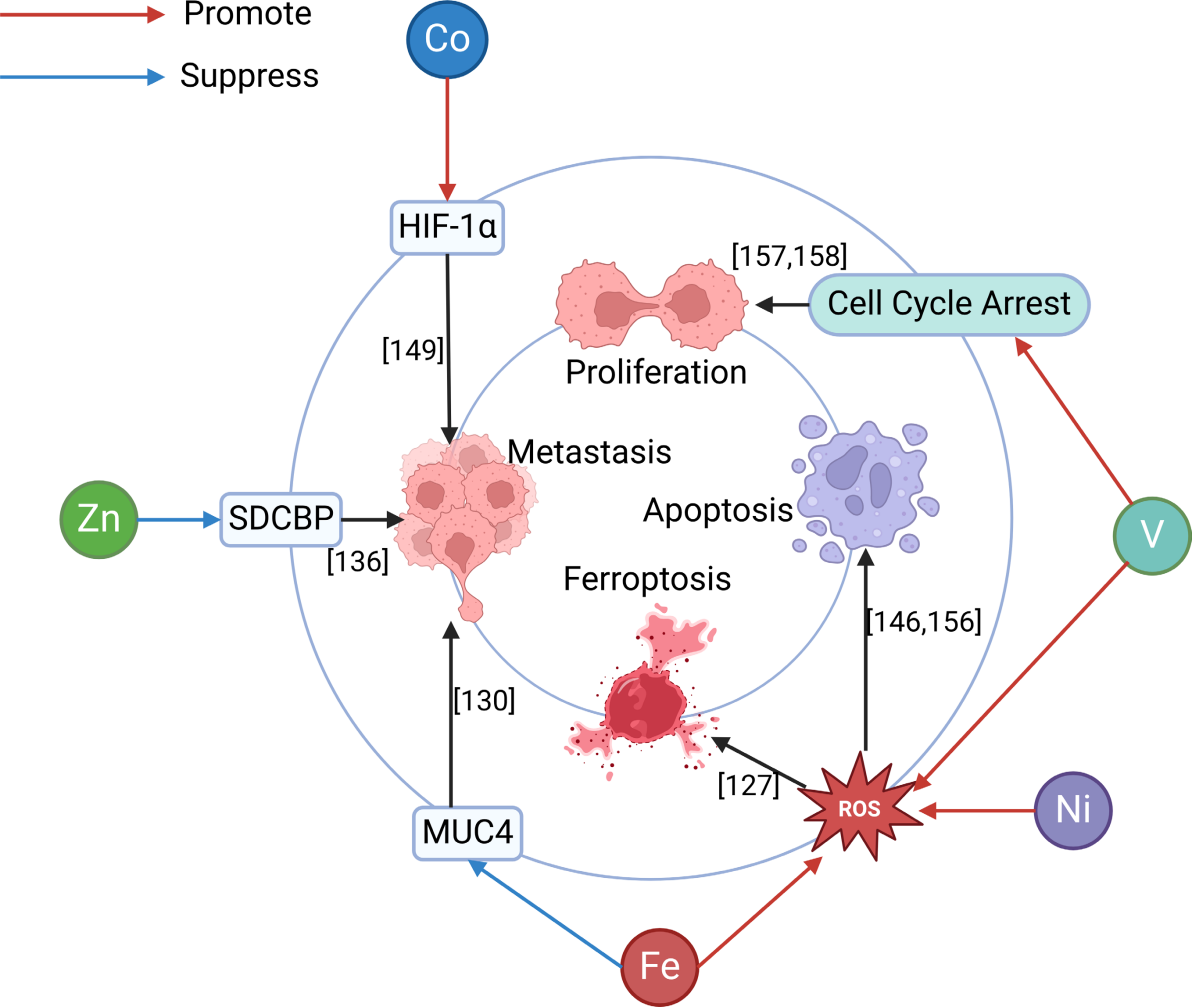
Mechanism of other trace elements in anti‐pancreatic cancer. This figure describes the APCA mechanisms associated with other trace elements, including inducing apoptosis, promoting ferroptosis, inhibiting metastasis, and inhibiting excessive proliferation. HIF‐1α, hypoxia‐inducible factor‐1α; MUC4, transmembrane glycoprotein; DCBP, syndecan binding protein.

In addition to the above elements, other related elements have also been studied (Figure 6). A study involving 78 patients with PCA and 416 healthy individuals indicated that patients with KRAS wild‐type PCA had higher concentrations of iron in their toenails than did those with KRAS mutant PCA.^83^ A controlled study involving 114 patients with PCA noted that patients with PCA occupationally exposed to Ahs had higher concentrations of iron in their toenails, but the difference was not statistically significant.^110^ Thus, the association between iron and the risk of PCA is not clear. Ferroptosis is a form of cell death in PCA that is caused by oxidative damage induced by excessive iron.^126^ In addition, iron oxide nanoparticles have been used to treat PCA through various methods, including photothermal therapy, immunotherapy^127^ and chemotherapy.^128^ Superparamagnetic iron oxide nanoparticles can be targeted to identify PCACs, enhance magnetic resonance imaging of PCA,^129^ and inhibit MUC4‐mediated metastasis in PCA,^130^ thus playing a role in the treatment of PCA. However, an animal study suggested that a high‐iron diet first promoted the release of 8‐hydroxy‐2′‐deoxyguanosine, activated the STING pathway, and ultimately led to macrophage infiltration and activation of KRAS‐driven PCA.^131^ Another study also noted that excessive iron decreases the expression of inhibitory P53 and leads to the occurrence of PCA.^132^ Iron is also a double‐edged sword for PCA, and maintaining iron homeostasis in the body is crucial to the health of the pancreas. A meta‐analysis involving six studies showed that high concentrations of zinc were significantly associated with a reduced risk of PCA.^133^ Syndecan binding protein (SDCBP) can promote tumor proliferation and metastasis.^134^ An animal study revealed that zinc pyrithione can inhibit the progression of PCA by inhibiting SDCBP.^135^ Like arsenic, cadmium is also a recognized human carcinogen.^136^ Several studies have reported that cadmium exposure increases the risk of PCA.^20^, ^83^, ^137^ Moreover, mechanistic studies have indicated that cadmium may cause PCA by altering mitochondrial function^138^, ^139^ and upregulating miR‐221,^140^ among which miR‐221 is one of the most carcinogenic miRNAs in PCA.^141^ However, a study noted that long‐term exposure to cadmium had no significant effect on the survival of patients with PCA.^142^ Lead is classified as a 2A carcinogen by the International Agency for Research on Cancer and has been associated with gastric, brain, kidney, lung,^143^ and PCA.^20^ However, the carcinogenic mechanism of lead in PCA has not been further studied. It has been reported that a high concentration of nickel is negatively correlated with the risk of PCA.^20^, ^83^ Nickel can change the expression concentrations of miRNAs, and miRNAs play important roles in the occurrence and progression of PCA.^144^ Nickel nanowires may be effective apoptotic agents for PCA treatment, and this effect is mediated by ROS.^145^ Chromium has been shown to increase the risk of PCA,^42^ but additional evidence is needed to support this finding. The organotin complex is cytotoxic to PCA,^146^ but its specific mechanism remains to be further studied. HIF‐1α counteracts the aggressiveness of PCA induced by hyperglycemia, and the concentration of HIF‐1α is positively correlated with the concentration of CoCl_2_, which may reduce the aggressiveness of PCA by increasing the expression of HIF‐1α.^147^ A study noted that CoCl_2_ can effectively induce apoptosis in PCACs by upregulating HIF‐1α.^148^ CoCl_2_ can construct an anoxic environment for PCACs,^149^ and hypoxia can activate HIF‐1α.^150^ CoCl_2_ may increase the expression of HIF‐1α by creating an anoxic environment, thus inhibiting PCA. Several studies have shown that molybdenum can damage pancreatic β cells and lead to diabetes,^139^, ^151^ while diabetic patients have a greater risk of PCA^152^; moreover, the association between molybdenum and PCA is worth studying. Higher concentrations of vanadium are associated with an increased risk of PCA.^38^ Interestingly, a number of studies have indicated that the vanadium complex has APCA activity, mainly through promoting apoptosis^153^, ^154^ and cell cycle arrest.^155^, ^156^ A cohort study in 2007 indicated that long‐term exposure to aluminum increased the risk of PCA,^157^ but the link between aluminum and PCA has yet to be determined.

## 7 CONCLUSION

During the development of PCA, selenium, manganese, and iron had dual effects on PCA, and they both promoted and inhibited the development of PCA. Maintaining homeostasis is very important for the health of the pancreas. Selenium, copper, and iron have shown unique value in the diagnosis of PCA by targeting imaging and improving diagnostic sensitivity. In vivo and in vitro experiments have verified that a variety of trace elements or their participating compounds have APCA activity, and the underlying mechanisms include promoting ferroptosis, inducing apoptosis, inhibiting metastasis, and inhibiting excessive proliferation. Selenium and manganese also have a certain impact on the prognosis of patients with PCA and can be used to construct prognostic models, even improving the prognosis of patients with PCA. However, most of the current literature reports are limited to only the research level, and there is still a lack of practical clinical application. However, additional studies are needed to verify the effectiveness and safety of these agents in clinical application. Therefore, evaluating the efficacy and safety of trace element therapy for the prevention, diagnosis and treatment of PCA is an important direction worth studying in the future.

## AUTHOR CONTRIBUTIONS

**Yao Yanjun:** Visualization (equal); writing – original draft (equal). **Zhuang Jing:** Writing – original draft (equal). **Song Yifei:** Writing – review and editing (equal). **Gu Gangzhao:** Visualization (equal). **Yan Chenxin:** Writing – review and editing (equal). **Wei Qiang:** Writing – review and editing (equal). **Yan Qiang:** Conceptualization (equal). **Han Shuwen:** Conceptualization (equal).

## FUNDING INFORMATION

This work was supported by the National Natural Science Foundation of China (Number: 82273339) and Medical and Health Research Project of Zhejiang Province (2022RC262).

## CONFLICT OF INTEREST STATEMENT

The authors declare that no potential conflicts of interest exist.

## Data Availability

Data sharing is not applicable to this article as no datasets were generated or analyzed during the current study.

## References

[1] Siegel RL , Miller KD , Fuchs HE , Jemal A . Cancer statistics, 2022. CA Cancer J Clin. 2022;72(1):7‐33. 35020204 10.3322/caac.21708

[2] Jiang D , Zhang L , Liu W , et al. Trends in cancer mortality in China from 2004 to 2018: a nationwide longitudinal study. Cancer Commun (Lond). 2021;41(10):1024‐1036. 34251754 10.1002/cac2.12195 PMC8504142

[3] GBD 2017 Pancreatic Cancer Collaborators . The global, regional, and national burden of pancreatic cancer and its attributable risk factors in 195 countries and territories, 1990–2017: a systematic analysis for the global burden of disease study 2017. Lancet Gastroenterol Hepatol. 2019;4:934‐947. 31648972 10.1016/S2468-1253(19)30347-4 PMC7026711

[4] Mizrahi JD , Surana R , Valle JW , Shroff RT . Pancreatic cancer. Lancet. 2020;395:2008‐2020. 32593337 10.1016/S0140-6736(20)30974-0

[5] Klein AP . Pancreatic cancer epidemiology: understanding the role of lifestyle and inherited risk factors. Nat Rev Gastroenterol Hepatol. 2021;18(7):493‐502. 34002083 10.1038/s41575-021-00457-x PMC9265847

[6] Andersen DK , Korc M , Petersen GM , et al. Diabetes, pancreatogenic diabetes, and pancreatic cancer. Diabetes. 2017;66:1103‐1110. 28507210 10.2337/db16-1477 PMC5399609

[7] Emsley J . Manganese the protector. Nat Chem. 2013;5(11):978. 24153378 10.1038/nchem.1783

[8] Ackland ML , Michalczyk AA . Zinc and infant nutrition. Arch Biochem Biophys. 2016;611:51‐57. 27317042 10.1016/j.abb.2016.06.011

[9] Olza J , Aranceta‐Bartrina J , González‐Gross M , et al. Reported dietary intake and food sources of zinc, selenium, and vitamins A, E and C in the Spanish population: findings from the ANIBES study. Nutrients. 2017;9(7):697. 28684689 10.3390/nu9070697 PMC5537812

[10] Tang D , Chen X , Kroemer G . Cuproptosis: a copper‐triggered modality of mitochondrial cell death. Cell Res. 2022;32(5):417‐418. 35354936 10.1038/s41422-022-00653-7 PMC9061796

[11] Castro Aguilar‐Tablada T , Navarro‐Alarcón M , Quesada Granados J , Samaniego Sánchez C , Rufián‐Henares JÁ , Nogueras‐Lopez F . Ulcerative colitis and Crohn's disease are associated with decreased serum selenium concentrations and increased cardiovascular risk. Nutrients. 2016;8(12):780. 27916926 10.3390/nu8120780 PMC5188435

[12] Rayman MP , Bath SC , Westaway J , et al. Selenium status in U.K. pregnant women and its relationship with hypertensive conditions of pregnancy. Br J Nutr. 2015;113(2):249‐258. 25571960 10.1017/S000711451400364X PMC4302388

[13] Gonzalez‐Alvarez MA , Hernandez‐Bonilla D , Plascencia‐Alvarez NI , Riojas‐Rodriguez H , Rosselli D . Environmental and occupational exposure to metals (manganese, mercury, iron) and Parkinson's disease in low and middle‐income countries: a narrative review. Rev Environ Health. 2021;37(1):1‐11. 33768768 10.1515/reveh-2020-0140

[14] Lu M , Deng P , Yang L , et al. Manganese overexposure induces Parkinson‐like symptoms, altered lipid signature and oxidative stress in C57BL/6 J mouse. Ecotoxicol Environ Saf. 2023;263:115238. 37441952 10.1016/j.ecoenv.2023.115238

[15] Cancarini A , Fostinelli J , Napoli L , Gilberti ME , Apostoli P , Semeraro F . Trace elements and diabetes: assessment of concentrations in tears and serum. Exp Eye Res. 2017;154:47‐52. 27984016 10.1016/j.exer.2016.10.020

[16] Amin MN , Siddiqui SA , Uddin MG , et al. Increased oxidative stress, altered trace elements, and macro‐minerals are associated with female obesity. Biol Trace Elem Res. 2020;197(2):384‐393. 31902098 10.1007/s12011-019-02002-z

[17] Cabré N , Luciano‐Mateo F , Arenas M , et al. Trace element concentrations in breast cancer patients. Breast. 2018;42:142‐149. 30296647 10.1016/j.breast.2018.09.005

[18] Kohzadi S , Sheikhesmaili F , Rahehagh R , et al. Evaluation of trace element concentration in cancerous and non‐cancerous tissues of human stomach. Chemosphere. 2017;184:747‐752. 28641226 10.1016/j.chemosphere.2017.06.071

[19] Sohrabi M , Gholami A , Azar MH , et al. Trace element and heavy metal concentrations in colorectal cancer: comparison between cancerous and non‐cancerous tissues. Biol Trace Elem Res. 2018;183(1):1‐8. 28795369 10.1007/s12011-017-1099-7

[20] Amaral AF , Porta M , Silverman DT , et al. Pancreatic cancer risk and concentrations of trace elements. Gut. 2012;61(11):1583‐1588. 22184070 10.1136/gutjnl-2011-301086 PMC3310963

[21] Chen QY , Costa M . PI3K/Akt/mTOR signaling pathway and the biphasic effect of arsenic in carcinogenesis. Mol Pharmacol. 2018;94(1):784‐792. doi:10.1124/mol.118.112268 Epub 2018 May 16. 29769245 PMC5994485

[22] Soza‐Ried C , Bustamante E , Caglevic C , Rolfo C , Sirera R , Marsiglia H . Oncogenic role of arsenic exposure in lung cancer: a forgotten risk factor. Crit Rev Oncol Hematol. 2019;139:128‐133. 30878179 10.1016/j.critrevonc.2019.01.012

[23] Wu R , Chen X , Wu H , et al. Nrf2 activation contributes to hepatic tumor‐augmenting effects of developmental arsenic exposure. Sci Total Environ. 2022;837:155685. 35523338 10.1016/j.scitotenv.2022.155685

[24] Brady DC , Crowe MS , Turski ML , et al. Copper is required for oncogenic BRAF signalling and tumorigenesis. Nature. 2014;509(7501):492‐496. 24717435 10.1038/nature13180 PMC4138975

[25] He H , Zou Z , Wang B , et al. Copper oxide nanoparticles induce oxidative DNA damage and cell death via copper ion‐mediated P38 MAPK activation in vascular endothelial cells. Int J Nanomedicine. 2020;15:3291‐3302. 32494130 10.2147/IJN.S241157 PMC7229313

[26] Su Y , Zhang X , Li S , Xie W , Guo J . Emerging roles of the copper‐CTR1 Axis in tumorigenesis. Mol Cancer Res. 2022;20(9):1339‐1353. 35604085 10.1158/1541-7786.MCR-22-0056

[27] Zhang S , Kang L , Dai X , et al. Manganese induces tumor cell ferroptosis through type‐I IFN dependent inhibition of mitochondrial dihydroorotate dehydrogenase. Free Radic Biol Med. 2022;193(Pt 1):202‐212. 36228830 10.1016/j.freeradbiomed.2022.10.004

[28] Lei H , Li Q , Pei Z , Liu L , Yang N , Cheng L . Nonferrous Ferroptosis inducer manganese Molybdate nanoparticles to enhance tumor immunotherapy. Small. 2023;19(45):e2303438. 37420331 10.1002/smll.202303438

[29] Tao W , Wang N , Ruan J , et al. Enhanced ROS‐boosted phototherapy against pancreatic cancer via Nrf2‐mediated stress‐defense pathway suppression and Ferroptosis induction. ACS Appl Mater Interfaces. 2022;14(5):6404‐6416. 35077153 10.1021/acsami.1c22861

[30] Jarosz M , Olbert M , Wyszogrodzka G , Młyniec K , Librowski T . Antioxidant and anti‐inflammatory effects of zinc. Zinc‐dependent NF‐κB signaling. Inflammopharmacology. 2017;25(1):11‐24. 28083748 10.1007/s10787-017-0309-4 PMC5306179

[31] Zhang X , He C , Yan R , et al. HIF‐1 dependent reversal of cisplatin resistance via anti‐oxidative nano selenium for effective cancer therapy. Chem Eng J. 2020;380:122540.

[32] Rataan AO , Geary SM , Zakharia Y , Rustum YM , Salem AK . Potential role of selenium in the treatment of cancer and viral infections. Int J Mol Sci. 2022;23(4):2215. 35216330 10.3390/ijms23042215 PMC8879146

[33] Faghfuri E , Yazdi MH , Mahdavi M , et al. Dose‐response relationship study of selenium nanoparticles as an immunostimulatory agent in cancer‐bearing mice. Arch Med Res. 2015;46(1):31‐37. 25604604 10.1016/j.arcmed.2015.01.002

[34] Filippini T , Fairweather‐Tait S , Vinceti M . Selenium and immune function: a systematic review and meta‐analysis of experimental human studies. Am J Clin Nutr. 2023;117(1):93‐110. 36789948 10.1016/j.ajcnut.2022.11.007

[35] Callejón‐Leblic B , Selma‐Royo M , Collado MC , Gómez‐Ariza JL , Abril N , García‐Barrera T . Untargeted gut metabolomics to delve the interplay between selenium supplementation and gut microbiota. J Proteome Res. 2022;21(3):758‐767. 34734730 10.1021/acs.jproteome.1c00411 PMC8902802

[36] Hu T , Li L , Hui G , et al. Selenium biofortification and its effect on multi‐element change in Auricularia auricular. Food Chem. 2019;295:206‐213. 31174751 10.1016/j.foodchem.2019.05.101

[37] Wang L , Wang J , Liu X , Liu Q , Zhang G , Liang L . Association between selenium intake and the risk of pancreatic cancer: a meta‐analysis of observational studies. Biosci Rep. 2016;36(5):e00395. 27623938 10.1042/BSR20160345 PMC5064452

[38] Chen J , Jiang W , Shao L , Zhong D , Wu Y , Cai J . Association between intake of antioxidants and pancreatic cancer risk: a meta‐analysis. Int J Food Sci Nutr. 2016;67(7):744‐753. 27356952 10.1080/09637486.2016.1197892

[39] Han X , Li J , Brasky TM , et al. Antioxidant intake and pancreatic cancer risk: the Vitamins and Lifestyle (VITAL) Study. Cancer. 2013;119(7):1314‐1320. 23280534 10.1002/cncr.27936 PMC3604041

[40] Banim PJ , Luben R , McTaggart A , et al. Dietary antioxidants and the aetiology of pancreatic cancer: a cohort study using data from food diaries and biomarkers. Gut. 2013;62(10):1489‐1496. 22826513 10.1136/gutjnl-2011-301908

[41] Lener MR , Scott RJ , Wiechowska‐Kozłowska A , et al. Serum concentrations of selenium and copper in patients diagnosed with pancreatic cancer. Cancer Res Treat. 2016;48(3):1056‐1064. 26727715 10.4143/crt.2015.282 PMC4946347

[42] Carrigan PE , Hentz JG , Gordon G , et al. Distinctive heavy metal composition of pancreatic juice in patients with pancreatic carcinoma. Cancer Epidemiol Biomarkers Prev. 2007;16(12):2656‐2663. 18086771 10.1158/1055-9965.EPI-07-0332

[43] Chatterjee S , Combs GF Jr , Chattopadhyay A , Stolzenberg‐Solomon R . Serum selenium and pancreatic cancer: a prospective study in the prostate, lung, colorectal and ovarian cancer trial cohort. Cancer Causes Control. 2019;30(5):457‐464. 30915619 10.1007/s10552-019-01147-5 PMC6752711

[44] Noè R , Inglese N , Romani P , et al. Organic selenium induces ferroptosis in pancreatic cancer cells. Redox Biol. 2023;68:102962. 38029455 10.1016/j.redox.2023.102962 PMC10698006

[45] Khalkar P , Díaz‐Argelich N , Antonio Palop J , Sanmartín C , Fernandes AP . Novel Methylselenoesters induce programed cell death via Entosis in pancreatic cancer cells. Int J Mol Sci. 2018;19(10):2849. 30241340 10.3390/ijms19102849 PMC6213452

[46] Moro CF , Selvam AK , Ghaderi M , et al. Drug‐induced tumor‐specific cytotoxicity in a whole tissue ex vivo model of human pancreatic ductal adenocarcinoma. Front Oncol. 2022;18(12):965182. 10.3389/fonc.2022.965182 PMC9436406 36059619

[47] Geismann C , Hauser C , Grohmann F , et al. NF‐κB/RelA controlled A20 limits TRAIL‐induced apoptosis in pancreatic cancer. Cell Death Dis. 2023;14(1):3. 36596765 10.1038/s41419-022-05535-9 PMC9810737

[48] Karelia DN , Kim S , Pandey MK , et al. Novel Seleno‐Aspirinyl compound AS‐10 induces apoptosis, G1 arrest of pancreatic ductal adenocarcinoma cells, inhibits their NF‐κB signaling, and synergizes with gemcitabine cytotoxicity. Int J Mol Sci. 2021;22(9):4966. 34067020 10.3390/ijms22094966 PMC8124556

[49] He SJ , Cao J , Li YS , et al. CdSe/ZnS quantum dots induce photodynamic effects and cytotoxicity in pancreatic cancer cells. World J Gastroenterol. 2016;22(21):5012‐5022. 27275093 10.3748/wjg.v22.i21.5012 PMC4886376

[50] Wooten DJ , Sinha I , Sinha R . Selenium induces pancreatic cancer cell death alone and in combination with gemcitabine. Biomedicine. 2022;10(1):149. 10.3390/biomedicines10010149 PMC8773897 35052828

[51] Teng T , Lin R , Lin Z , et al. Photothermal augment stromal disrupting effects for enhanced Abraxane synergy chemotherapy in pancreatic cancer PDX mode. Biomater Sci. 2020;8(12):3278‐3285. 32355947 10.1039/d0bm00549e

[52] Ge XL , Huang B , Zhang ZL , et al. Glucose‐functionalized near‐infrared Ag2Se quantum dots with renal excretion ability for long‐term in vivo tumor imaging. J Mater Chem B. 2019;7(38):5782‐5788. 31482937 10.1039/c9tb01112a

[53] Pfister C , Schoenemann J . Selenium in cancer rehabilitation‐a retrospective study from a specialized clinic. Nutrients. 2023;15(17):3827. 37686861 10.3390/nu15173827 PMC10490249

[54] Tulinska J , Mikusova ML , Liskova A , et al. Copper oxide nanoparticles stimulate the immune response and decrease antioxidant defense in mice after six‐week inhalation. Front Immunol. 2022;13:874253. 35547729 10.3389/fimmu.2022.874253 PMC9082266

[55] Flemming A . Copper boosts pro‐inflammatory state of macrophages. Nat Rev Immunol. 2023;23(6):344. 10.1038/s41577-023-00889-3 PMC10176277 37173540

[56] Agga GE , Scott HM , Amachawadi RG , et al. Effects of chlortetracycline and copper supplementation on antimicrobial resistance of fecal Escherichia coli from weaned pigs. Prev Vet Med. 2014;114(3–4):231‐246. 24655578 10.1016/j.prevetmed.2014.02.010

[57] Schilling K , Larner F , Saad A , et al. Urine metallomics signature as an indicator of pancreatic cancer. Metallomics. 2020;12(5):752‐757. 32211672 10.1039/d0mt00061b

[58] Xue Q , Yan D , Chen X , et al. Copper‐dependent autophagic degradation of GPX4 drives ferroptosis. Autophagy. 2023;19(7):1982‐1996. 36622894 10.1080/15548627.2023.2165323 PMC10283421

[59] Gou Y , Chen M , Li S , et al. Dithiocarbazate‐copper complexes for bioimaging and treatment of pancreatic cancer. J Med Chem. 2021;64(9):5485‐5499. 33861929 10.1021/acs.jmedchem.0c01936

[60] Hurtado M , Prokai L , Sankpal UT , et al. Next generation sequencing and functional pathway analysis to understand the mechanism of action of copper‐tolfenamic acid against pancreatic cancer cells. Process Biochem. 2020;89:155‐164. 32719579 10.1016/j.procbio.2019.10.022 PMC7384693

[61] Li T , Su L , Lei Y , Liu X , Zhang Y , Liu X . DDIT3 and KAT2A proteins regulate TNFRSF10A and TNFRSF10B expression in endoplasmic reticulum stress‐mediated apoptosis in human lung cancer cells. J Biol Chem. 2015;290(17):11108‐11118. 25770212 10.1074/jbc.M115.645333 PMC4409269

[62] Kang SJ , Rhee WJ . Silkworm storage protein 1 inhibits autophagy‐mediated apoptosis. Int J Mol Sci. 2019;20(2):318. 30646576 10.3390/ijms20020318 PMC6359030

[63] Gourisankar S , Krokhotin A , Ji W , et al. Rewiring cancer drivers to activate apoptosis. Nature. 2023;620(7973):417‐425. 37495688 10.1038/s41586-023-06348-2 PMC10749586

[64] López‐Malpartida AV , Ludeña MD , Varela G , García PJ . Differential ErbB receptor expression and intracellular signaling activity in lung adenocarcinomas and squamous cell carcinomas. Lung Cancer. 2009;65(1):25‐33. 19046792 10.1016/j.lungcan.2008.10.009

[65] Tian Z , Ou G , Su M , et al. TIMP1 derived from pancreatic cancer cells stimulates Schwann cells and promotes the occurrence of perineural invasion. Cancer Lett. 2022;546:215863. 35961511 10.1016/j.canlet.2022.215863

[66] Masuri S , Moráň L , Vesselá T , et al. A novel heteroleptic Cu(II)‐phenanthroline‐UDCA complex as lipoxygenase inhibitor and ER‐stress inducer in cancer cell lines. J Inorg Biochem. 2023;246:112301. 37392615 10.1016/j.jinorgbio.2023.112301

[67] Hossan MS , Break MKB , Bradshaw TD , et al. Novel semi‐synthetic Cu (II)‐Cardamonin complex exerts potent anticancer activity against triple‐negative breast and pancreatic cancer cells via inhibition of the Akt signaling pathway. Molecules. 2021;26(8):2166. 33918814 10.3390/molecules26082166 PMC8069646

[68] Mo X , Shen X , Mo X , et al. CEMIP promotes small cell lung cancer proliferation by activation of glutamine metabolism via FBXW7/c‐Myc‐dependent axis. Biochem Pharmacol. 2023;209:115446. 36746261 10.1016/j.bcp.2023.115446

[69] Del Bello F , Pellei M , Bagnarelli L , et al. Cu(I) and Cu(II) complexes based on Lonidamine‐conjugated ligands designed to promote synergistic antitumor effects. Inorg Chem. 2022;61(12):4919‐4937. 35285628 10.1021/acs.inorgchem.1c03658 PMC8965879

[70] Li X , Xu H , Li C , et al. Zinc‐doped copper oxide nanocomposites inhibit the growth of pancreatic cancer by inducing autophagy through AMPK/mTOR pathway. Front Pharmacol. 2019;10:319. 31001120 10.3389/fphar.2019.00319 PMC6454023

[71] Roy J , Kyani A , Hanafi M , et al. Design and synthesis of orally active Quinolyl Pyrazinamides as sigma 2 receptor ligands for the treatment of pancreatic cancer. J Med Chem. 2023;66(3):1990‐2019. 36692906 10.1021/acs.jmedchem.2c01769 PMC12167926

[72] Fantoni NZ , Molphy Z , O'Carroll S , et al. Polypyridyl‐based copper Phenanthrene complexes: combining stability with enhanced DNA recognition. Chemistry. 2021;27(3):971‐983. 32519773 10.1002/chem.202001996

[73] Arjmand F , Sharma S , Parveen S , Toupet L , Yu Z , Cowan JA . Copper (ii) l/d‐valine‐(1,10‐phen) complexes target human telomeric G‐quadruplex motifs and promote site‐specific DNA cleavage and cellular cytotoxicity. Dalton Trans. 2020;49(28):9888‐9899. 32638779 10.1039/d0dt01527j PMC7433390

[74] Yoshii Y , Tashima H , Iwao Y , et al. Immuno‐OpenPET: a novel approach for early diagnosis and image‐guided surgery for small resectable pancreatic cancer. Sci Rep. 2020;10(1):4143. 32157106 10.1038/s41598-020-61056-5 PMC7064510

[75] Yu Q , Zhou J , Liu Y , et al. DNAzyme‐mediated Cascade Nanoreactor for Cuproptosis‐promoted pancreatic cancer synergistic therapy. Adv Healthc Mater. 2023;12(28):e2301429. 37548109 10.1002/adhm.202301429

[76] Benguigui M , Weitz IS , Timaner M , et al. Copper oxide nanoparticles inhibit pancreatic tumor growth primarily by targeting tumor initiating cells. Sci Rep. 2019;9(1):12613. 31471546 10.1038/s41598-019-48959-8 PMC6717199

[77] Zhang X , Detering L , Sultan D , et al. CC chemokine receptor 2‐targeting copper nanoparticles for positron emission tomography‐guided delivery of gemcitabine for pancreatic ductal adenocarcinoma. ACS Nano. 2021;15(1):1186‐1198. 33406361 10.1021/acsnano.0c08185 PMC7846978

[78] Zhang H , Chen K , Guo K , et al. Multimodal imaging‐guided Photoimmunotherapy of pancreatic cancer by Organosilica Nanomedicine. Adv Healthc Mater. 2023;13:e2302195. 37792547 10.1002/adhm.202302195

[79] Geng R , Ke N , Wang Z , et al. Copper deprivation enhances the chemosensitivity of pancreatic cancer to rapamycin by mTORC1/2 inhibition. Chem Biol Interact. 2023;382:110546. 37290678 10.1016/j.cbi.2023.110546

[80] Song G , Dong H , Ma D , et al. Tetrahedral framework nucleic acid delivered RNA therapeutics significantly attenuate pancreatic cancer progression via inhibition of CTR1‐dependent copper absorption. ACS Appl Mater Interfaces. 2021;13(39):46334‐46342. 34549583 10.1021/acsami.1c13091

[81] Giles BH , Mann KK . Arsenic as an immunotoxicant. Toxicol Appl Pharmacol. 2022;454:116248. 36122737 10.1016/j.taap.2022.116248

[82] Griggs JL , Chi L , Hanley NM , et al. Bioaccessibility of arsenic from contaminated soils and alteration of the gut microbiome in an in vitro gastrointestinal model. Environ Pollut. 2022;309:119753. 35835276 10.1016/j.envpol.2022.119753 PMC9667710

[83] Gómez‐Tomás Á , Pumarega J , Alguacil J , et al. Concentrations of trace elements and KRAS mutations in pancreatic ductal adenocarcinoma. Environ Mol Mutagen. 2019;60(8):693‐703. 31066938 10.1002/em.22296 PMC6786909

[84] Liu‐Mares W , Mackinnon JA , Sherman R , et al. Pancreatic cancer clusters and arsenic‐contaminated drinking water wells in Florida. BMC Cancer. 2013;13:111. 23510413 10.1186/1471-2407-13-111 PMC3600048

[85] Zeng L , Li J , Wang Y , et al. Combination of siRNA‐directed Kras oncogene silencing and arsenic‐induced apoptosis using a nanomedicine strategy for the effective treatment of pancreatic cancer. Nanomedicine. 2014;10(2):463‐472. 24028894 10.1016/j.nano.2013.08.007

[86] Yu S , Wu N , Zhu J , Liu Y , Han J . Pyrrolidine Dithiocarbamate facilitates arsenic trioxide against pancreatic cancer via perturbing ubiquitin‐proteasome pathway. Cancer Manag Res. 2020;12:13149‐13159. 33376406 10.2147/CMAR.S278674 PMC7764808

[87] Leng RP , Lin Y , Ma W , et al. Pirh2, a p53‐induced ubiquitin‐protein ligase, promotes p53 degradation. Cell. 2003;112(6):779‐791. 12654245 10.1016/s0092-8674(03)00193-4

[88] Xu C , Wang X , Zhou Y , et al. Synergy between arsenic trioxide and JQ1 on autophagy in pancreatic cancer. Oncogene. 2019;38(47):7249‐7265. 31420604 10.1038/s41388-019-0930-3

[89] Ahmad IM , Dafferner AJ , Salloom RJ , Abdalla MY . Heme Oxygenase‐1 inhibition modulates autophagy and augments arsenic trioxide cytotoxicity in pancreatic cancer cells. Biomedicine. 2023;11(9):2580. 10.3390/biomedicines11092580 PMC10526552 37761021

[90] Lang M , Wang X , Wang H , et al. Arsenic trioxide plus PX‐478 achieves effective treatment in pancreatic ductal adenocarcinoma. Cancer Lett. 2016;378(2):87‐96. 27212442 10.1016/j.canlet.2016.05.016

[91] Baumgartner M , Sturlan S , Roth E , Wessner B , Bachleitner‐Hofmann T . Enhancement of arsenic trioxide‐mediated apoptosis using docosahexaenoic acid in arsenic trioxide‐resistant solid tumor cells. Int J Cancer. 2004;112(4):707‐712. 15382055 10.1002/ijc.20462

[92] Tian Z , Tan Y , Lin X , et al. Arsenic trioxide sensitizes pancreatic cancer cells to gemcitabine through downregulation of the TIMP1/PI3K/AKT/mTOR axis. Transl Res. 2023;255:66‐76. 36400307 10.1016/j.trsl.2022.11.007

[93] Zhao Y , Yao H , Yang K , et al. Arsenic trioxide‐loaded nanoparticles enhance the chemosensitivity of gemcitabine in pancreatic cancer via the reversal of pancreatic stellate cell desmoplasia by targeting the AP4/galectin‐1 pathway. Biomater Sci. 2022;10(20):5989‐6002. 36052559 10.1039/d2bm01039a

[94] Gao J , Wang G , Wu J , Zuo Y , Zhang J , Jin X . Skp2 expression is inhibited by arsenic trioxide through the upregulation of miRNA‐330‐5p in pancreatic cancer cells. Mol Ther Oncolytics. 2019;12:214‐223. 30847385 10.1016/j.omto.2019.01.006 PMC6389777

[95] Jin J , Harper JW . A license to kill: transcriptional activation and enhanced turnover of Myc by the SCF(kp2) ubiquitin ligase. Cancer Cell. 2003;3(6):517‐518. 12842079 10.1016/s1535-6108(03)00145-4

[96] Ghanbari A , Cheraghzadeh Z , Mahmoudi R , Zibara K , Hosseini E . GLI inhibitors overcome Erlotinib resistance in human pancreatic cancer cells by modulating E‐cadherin. J Chemother. 2019;31(3):141‐149. 30983542 10.1080/1120009X.2019.1584422

[97] Tang R , Zhu J , Liu Y , Wu N , Han J . Formulation comprising arsenic trioxide and Dimercaprol enhances Radiosensitivity of pancreatic cancer xenografts. Technol Cancer Res Treat. 2021;20:15330338211036324. 34433326 10.1177/15330338211036324 PMC8404670

[98] Li C , Heidt DG , Dalerba P , et al. Identification of pancreatic cancer stem cells. Cancer Res. 2007;67(3):1030‐1037. 17283135 10.1158/0008-5472.CAN-06-2030

[99] Ding W , Zhang L , Kim S , et al. Arsenic sulfide as a potential anti cancer drug. Mol Med Rep. 2015;11(2):968‐974. 25371265 10.3892/mmr.2014.2838 PMC4262477

[100] Yang MH , Kim HT , Lee KT , et al. KML001 inhibits cell proliferation and invasion in pancreatic cancer cells through suppression of NF‐κB and VEGF‐C. Anticancer Res. 2014;34(7):3469‐3474. 24982355

[101] Yang MH , Lee KT , Yang S , Lee JK , Lee KH , Rhee JC . KML001 enhances anticancer activity of gemcitabine against pancreatic cancer cells. Anticancer Res. 2015;35(1):183‐189. 25550550

[102] Mucciolo G , Araos Henríquez J , Jihad M , et al. EGFR‐activated myofibroblasts promote metastasis of pancreatic cancer. Cancer Cell. 2024;42:101‐118.e11. 38157863 10.1016/j.ccell.2023.12.002

[103] Han Y , Ma L , Zhao L , Feng W , Zheng X . Rosmarinic inhibits cell proliferation, invasion and migration via up‐regulating miR‐506 and suppressing MMP2/16 expression in pancreatic cancer. Biomed Pharmacother. 2019;115:108878. 31060006 10.1016/j.biopha.2019.108878

[104] Viale A , Pettazzoni P , Lyssiotis CA , et al. Oncogene ablation‐resistant pancreatic cancer cells depend on mitochondrial function. Nature. 2014;514(7524):628‐632. 25119024 10.1038/nature13611 PMC4376130

[105] Ashton TM , McKenna WG , Kunz‐Schughart LA , Higgins GS . Oxidative phosphorylation as an emerging target in cancer therapy. Clin Cancer Res. 2018;24(11):2482‐2490. 29420223 10.1158/1078-0432.CCR-17-3070

[106] Zhang R , Wang C , Guan Y , et al. Manganese salts function as potent adjuvants. Cell Mol Immunol. 2021;18(5):1222‐1234. 33767434 10.1038/s41423-021-00669-w PMC8093200

[107] Chi L , Gao B , Bian X , Tu P , Ru H , Lu K . Manganese‐induced sex‐specific gut microbiome perturbations in C57BL/6 mice. Toxicol Appl Pharmacol. 2017;331:142‐153. 28610994 10.1016/j.taap.2017.06.008 PMC5653225

[108] Pushalkar S , Hundeyin M , Daley D , et al. The pancreatic cancer microbiome promotes Oncogenesis by induction of innate and adaptive immune suppression. Cancer Discov. 2018;8(4):403‐416. 29567829 10.1158/2159-8290.CD-17-1134 PMC6225783

[109] Hutchens S , Jursa TP , Melkote A , Grant SM , Smith DR , Mukhopadhyay S . Hepatic and intestinal manganese excretion are both required to regulate brain manganese during elevated manganese exposure. Am J Physiol Gastrointest Liver Physiol. 2023;325(3):G251‐G264. 37461848 10.1152/ajpgi.00047.2023 PMC10511180

[110] Camargo J , Pumarega JA , Alguacil J , et al. Toenail concentrations of trace elements and occupational history in pancreatic cancer. Environ Int. 2019;127:216‐225. 30928845 10.1016/j.envint.2019.03.037

[111] Chen H , Cui Z , Lu W , et al. Association between serum manganese concentrations and diabetes in Chinese adults with hypertension. J Clin Hypertens (Greenwich). 2022;24(7):918‐927. 35748116 10.1111/jch.14520 PMC9278588

[112] Wang Y , Yang C , Hu R , et al. Assembling Mn:ZnSe quantum dots‐siRNA nanoplexes for gene silencing in tumor cells. Biomater Sci. 2015;3(1):192‐202. 26214202 10.1039/c4bm00306c

[113] Tong S , Yu Z , Yin F , et al. Manganese‐based Prussian blue nanoparticles inhibit tumor proliferation and migration via the MAPK pathway in pancreatic cancer. Front Chem. 2022;24(10):1026924. 10.3389/fchem.2022.1026924 PMC9638070 36353142

[114] Guan G , Liu H , Xu J , et al. Ultrasmall PtMn nanoparticles as sensitive manganese release modulator for specificity cancer theranostics. J Nanobiotechnology. 2023;21(1):434. 37980476 10.1186/s12951-023-02172-y PMC10657629

[115] Pandit H , Zhang W , Li Y , et al. Manganese superoxide dismutase expression is negatively associated with microRNA‐301a in human pancreatic ductal adenocarcinoma. Cancer Gene Ther. 2015;22(10):481‐486. 26384137 10.1038/cgt.2015.46 PMC4670085

[116] Cheng G , Lanza‐Jacoby S . Metformin decreases growth of pancreatic cancer cells by decreasing reactive oxygen species: role of NOX4. Biochem Biophys Res Commun. 2015;465(1):41‐46. 26225747 10.1016/j.bbrc.2015.07.118

[117] McCormack DE , Mannal P , McDonald D , Tighe S , Hanson J , McFadden D . Genomic analysis of pterostilbene predicts its antiproliferative effects against pancreatic cancer in vitro and in vivo. J Gastrointest Surg. 2012;16(6):1136‐1143. 22450950 10.1007/s11605-012-1869-7 PMC4237162

[118] Liu F , Xiang Q , Luo Y , et al. A hybrid nanopharmaceutical for specific‐amplifying oxidative stress to initiate a cascade of catalytic therapy for pancreatic cancer. J Nanobiotechnology. 2023;21(1):165. 37221521 10.1186/s12951-023-01932-0 PMC10207777

[119] Cieslak JA , Strother RK , Rawal M , et al. Manganoporphyrins and ascorbate enhance gemcitabine cytotoxicity in pancreatic cancer. Free Radic Biol Med. 2015;83:227‐237. 25725418 10.1016/j.freeradbiomed.2015.02.018 PMC4441864

[120] Tovmasyan A , Sampaio RS , Boss MK , et al. Anticancer therapeutic potential of Mn porphyrin/ascorbate system. Free Radic Biol Med. 2015;89:1231‐1247. 26496207 10.1016/j.freeradbiomed.2015.10.416 PMC4684782

[121] Liu G , Liu M , Li X , et al. Peroxide‐simulating and GSH‐depleting Nanozyme for enhanced Chemodynamic/photodynamic therapy via induction of multisource ROS. ACS Appl Mater Interfaces. 2023;15(41):47955‐47968. 37812458 10.1021/acsami.3c09873

[122] Alexander MS , O'Leary BR , Wilkes JG , et al. Enhanced pharmacological ascorbate oxidation Radiosensitizes pancreatic cancer. Radiat Res. 2019;191(1):43‐51. 30376411 10.1667/RR15189.1 PMC6441967

[123] Shin SW , Jung W , Choi C , et al. Fucoidan‐manganese dioxide nanoparticles potentiate radiation therapy by Co‐targeting tumor hypoxia and angiogenesis. Mar Drugs. 2018;16(12):510. 30558324 10.3390/md16120510 PMC6316049

[124] Sishc BJ , Ding L , Nam TK , et al. Avasopasem manganese synergizes with hypofractionated radiation to ablate tumors through the generation of hydrogen peroxide. Sci Transl Med. 2021;13(593):eabb3768. 33980575 10.1126/scitranslmed.abb3768 PMC8314936

[125] Xiao H , Li X , Li B , et al. Sono‐promoted drug penetration and extracellular matrix modulation potentiate sonodynamic therapy of pancreatic ductal adenocarcinoma. Acta Biomater. 2023;161:265‐274. 36893956 10.1016/j.actbio.2023.02.038

[126] Tang D , Kroemer G . Ferroptosis. Curr Biol. 2020;30(21):R1292‐R1297. 33142092 10.1016/j.cub.2020.09.068

[127] Wang M , Li Y , Wang M , et al. Synergistic interventional photothermal therapy and immunotherapy using an iron oxide nanoplatform for the treatment of pancreatic cancer. Acta Biomater. 2022;15(138):453‐462. 10.1016/j.actbio.2021.10.048 PMC10960566 34757232

[128] Qi G , Shi G , Wang S , et al. A novel pH‐responsive iron oxide Core‐Shell magnetic mesoporous silica nanoparticle (M‐MSN) system encapsulating doxorubicin (DOX) and glucose oxidase (Gox) for pancreatic cancer treatment. Int J Nanomedicine. 2023;30(18):7133‐7147. 10.2147/IJN.S436253 PMC10695029 38054080

[129] Pu Y , Ke H , Wu C , et al. Superparamagnetic iron oxide nanoparticles target BxPC‐3 cells and silence MUC4 for the treatment of pancreatic cancer. Biochim Biophys Acta Gen Subj. 2023;1867(9):130383. 37236323 10.1016/j.bbagen.2023.130383

[130] Senapati S , Gnanapragassam VS , Moniaux N , Momi N , Batra SK . Role of MUC4‐NIDO domain in the MUC4‐mediated metastasis of pancreatic cancer cells. Oncogene. 2012;31(28):3346‐3356. 22105367 10.1038/onc.2011.505 PMC3298579

[131] Dai E , Han L , Liu J , et al. Ferroptotic damage promotes pancreatic tumorigenesis through a TMEM173/STING‐dependent DNA sensor pathway. Nat Commun. 2020;11(1):6339. 33311482 10.1038/s41467-020-20154-8 PMC7732843

[132] Bhutia YD , Ogura J , Grippo PJ , et al. Chronic exposure to excess iron promotes EMT and cancer via p53 loss in pancreatic cancer. Asian J Pharm Sci. 2020;15(2):237‐251. 32373202 10.1016/j.ajps.2020.02.003 PMC7193456

[133] Li L , Gai X . The association between dietary zinc intake and risk of pancreatic cancer: a meta‐analysis. Biosci Rep. 2017;37(3):BSR20170155. 28428431 10.1042/BSR20170155 PMC5463257

[134] Das SK , Maji S , Wechman SL , et al. MDA‐9/Syntenin (SDCBP): novel gene and therapeutic target for cancer metastasis. Pharmacol Res. 2020;155:104695. 32061839 10.1016/j.phrs.2020.104695 PMC7551653

[135] Liu J , Bai W , Zhou T , et al. SDCBP promotes pancreatic cancer progression by preventing YAP1 from β‐TrCP‐mediated proteasomal degradation. Gut. 2023;72(9):1722‐1737. 36828627 10.1136/gutjnl-2022-327492

[136] Tinkov AA , Filippini T , Ajsuvakova OP , et al. Cadmium and atherosclerosis: a review of toxicological mechanisms and a meta‐analysis of epidemiologic studies. Environ Res. 2018;162:240‐260. 29358116 10.1016/j.envres.2018.01.008

[137] Djordjevic VR , Wallace DR , Schweitzer A , et al. Environmental cadmium exposure and pancreatic cancer: evidence from case control, animal and in vitro studies. Environ Int. 2019;128:353‐361. 31078004 10.1016/j.envint.2019.04.048

[138] Wallace DR , Spandidos DA , Tsatsakis A , Schweitzer A , Djordjevic V , Djordjevic AB . Potential interaction of cadmium chloride with pancreatic mitochondria: implications for pancreatic cancer. Int J Mol Med. 2019;44(1):145‐156. 31115542 10.3892/ijmm.2019.4204 PMC6559323

[139] Elmorsy E , Al‐Ghafari A , Al Doghaither H , Ghulam J . Effects of environmental metals on mitochondrial bioenergetics of the CD‐1 mice pancreatic beta‐cells. Toxicol In Vitro. 2021;70:105015. 33038468 10.1016/j.tiv.2020.105015

[140] Mortoglou M , Buha Djordjevic A , Djordjevic V , et al. Role of microRNAs in response to cadmium chloride in pancreatic ductal adenocarcinoma. Arch Toxicol. 2022;96(2):467‐485. 34905088 10.1007/s00204-021-03196-9 PMC8837568

[141] Uysal‐Onganer P , D'Alessio S , Mortoglou M , Kraev I , Lange S . Peptidylarginine deiminase inhibitor application, using Cl‐Amidine, PAD2, PAD3 and PAD4 isozyme‐specific inhibitors in pancreatic cancer cells, reveals roles for PAD2 and PAD3 in cancer invasion and modulation of extracellular vesicle signatures. Int J Mol Sci. 2021;22(3):1396. 33573274 10.3390/ijms22031396 PMC7866560

[142] Porta M , Pumarega J , Amaral AFS , et al. Influence of KRAS mutations, persistent organic pollutants, and trace elements on survival from pancreatic ductal adenocarcinoma. Environ Res. 2020;190:109781. 32791343 10.1016/j.envres.2020.109781 PMC7689512

[143] Steenland K , Barry V , Anttila A , et al. Cancer incidence among workers with blood lead measurements in two countries. Occup Environ Med. 2019;76(9):603‐610. 31296664 10.1136/oemed-2019-105786

[144] Mortoglou M , Manić L , Buha Djordjevic A , et al. Nickel's role in pancreatic ductal adenocarcinoma: potential involvement of microRNAs. Toxics. 2022;10(3):148. 35324773 10.3390/toxics10030148 PMC8952337

[145] Hossain MZ , Kleve MG . Nickel nanowires induced and reactive oxygen species mediated apoptosis in human pancreatic adenocarcinoma cells. Int J Nanomedicine. 2011;6:1475‐1485. 21845039 10.2147/IJN.S21697 PMC3152467

[146] Balogová M , Sharma S , Cherek P , et al. Cytotoxic effects of halogenated tin phosphinoyldithioformate complexes against several cancer cell lines. Dalton Trans. 2022;51(34):13119‐13128. 35975724 10.1039/d2dt01127a

[147] Li W , Liu H , Qian W , et al. Hyperglycemia aggravates microenvironment hypoxia and promotes the metastatic ability of pancreatic cancer. Comput Struct Biotechnol J. 2018;16:479‐487. 30455857 10.1016/j.csbj.2018.10.006 PMC6232646

[148] Dai ZJ , Gao J , Ma XB , et al. Up‐regulation of hypoxia inducible factor‐1α by cobalt chloride correlates with proliferation and apoptosis in PC‐2 cells. J Exp Clin Cancer Res. 2012;31(1):28. 22453051 10.1186/1756-9966-31-28 PMC3359273

[149] Han S , Kim K , Thakkar N , Kim D , Lee W . Role of hypoxia inducible factor‐1α in the regulation of the cancer‐specific variant of organic anion transporting polypeptide 1B3 (OATP1B3), in colon and pancreatic cancer. Biochem Pharmacol. 2013;86(6):816‐823. 23924606 10.1016/j.bcp.2013.07.020

[150] Korbecki J , Simińska D , Gąssowska‐Dobrowolska M , et al. Chronic and cycling hypoxia: drivers of cancer chronic inflammation through HIF‐1 and NF‐κB activation: a review of the molecular mechanisms. Int J Mol Sci. 2021;22(19):10701. 34639040 10.3390/ijms221910701 PMC8509318

[151] Al Doghaither H , Elmorsy E , Al‐Ghafari A , Ghulam J . Roles of oxidative stress, apoptosis, and inflammation in metal‐induced dysfunction of beta pancreatic cells isolated from CD1 mice. Saudi J Biol Sci. 2021;28(1):651‐663. 33424352 10.1016/j.sjbs.2020.10.056 PMC7785459

[152] Sharma S , Tapper WJ , Collins A , Hamady ZZR . Predicting pancreatic cancer in the UK biobank cohort using polygenic risk scores and diabetes mellitus. Gastroenterology. 2022;162(6):1665‐1674.e2. 35065983 10.1053/j.gastro.2022.01.016

[153] Griffin E , Levina A , Lay PA . Vanadium(V) tris‐3,5‐di‐tert‐butylcatecholato complex: links between speciation and anti‐proliferative activity in human pancreatic cancer cells. J Inorg Biochem. 2019;201:110815. 31520878 10.1016/j.jinorgbio.2019.110815

[154] Kowalski S , Tesmar A , Sikorski A , Inkielewicz‐Stępniak I . Oxidovanadium(IV) complex disrupts mitochondrial membrane potential and induces apoptosis in pancreatic cancer cells. Anti Cancer Agents Med Chem. 2021;21(1):71‐83. 10.2174/1871520620666200624145217 32579508

[155] Wu JX , Hong YH , Yang XG . Bis(acetylacetonato)‐oxidovanadium(IV) and sodium metavanadate inhibit cell proliferation via ROS‐induced sustained MAPK/ERK activation but with elevated AKT activity in human pancreatic cancer AsPC‐1 cells. J Biol Inorg Chem. 2016;21(8):919‐929. 27614430 10.1007/s00775-016-1389-0

[156] Kowalski S , Wyrzykowski D , Hac S , Rychlowski M , Radomski MW , Inkielewicz‐Stepniak I . New Oxidovanadium(IV) coordination complex containing 2‐Methylnitrilotriacetate ligands induces cell cycle arrest and autophagy in human pancreatic ductal adenocarcinoma cell lines. Int J Mol Sci. 2019;20(2):261. 30634697 10.3390/ijms20020261 PMC6358955

[157] Gibbs GW , Sevigny M . Mortality and cancer experience of Quebec aluminum reduction plant workers, part 4: cancer incidence. J Occup Environ Med. 2007;49(12):1351‐1366. 18231082 10.1097/JOM.0b013e318156ecbc

